# Association and Incremental Discriminative Value of Four Estimated Muscle-Related Indices for Progression from Prediabetes to Type 2 Diabetes Mellitus: A Retrospective Cohort Study with up to 10 Years of Follow-Up

**DOI:** 10.3390/jcm15207828

**Published:** 2026-10-10

**Authors:** Xinrui Cao, Yan He, Zhihao Ni, Guoli Shen, Leiwen Tang, Ting Sun

**Affiliations:** Department of Nursing, The Second Affiliated Hospital of Zhejiang University School of Medicine, Hangzhou 310009, China; 22418405@zju.edu.cn (X.C.);

**Keywords:** prediabetes, type 2 diabetes mellitus, estimated muscle-related indices, cohort study

## Abstract

**Objective:** This study examined the associations of four estimated muscle-related indices (height-adjusted appendicular skeletal muscle mass [ASM/height^2^], weight-adjusted ASM [ASM/weight], predicted skeletal muscle mass index [pSMI], and creatinine-to-cystatin C ratio [CCR]) with progression from prediabetes to type 2 diabetes mellitus (T2DM) and compared their preliminary predictive performance. **Methods:** This retrospective cohort included 927 Chinese adults with prediabetes. Incident T2DM was the primary endpoint. Associations were assessed using Cox regression, dose–response relationships using restricted cubic splines, and incremental discrimination using time-dependent AUC with bootstrap internal validation. Subgroup analyses were performed by sex and body mass index (BMI). Sensitivity analyses additionally adjusted for age and HbA1c and excluded early incident T2DM cases. **Results:** During a median follow-up of 4.23 years, 211 participants (22.76%) developed T2DM. In the primary multivariable model, ASM/weight (HR = 0.54 [0.34, 0.86], *p* = 0.009) and pSMI (HR = 0.73 [0.53, 0.99], *p* = 0.041) were inversely associated with T2DM. However, only ASM/weight remained significant after false discovery rate correction, and all associations were attenuated after additional adjustment for age and HbA1c. After excluding early incident T2DM cases, the association of ASM/weight was no longer significant. CCR showed a U-shaped association. The incremental discriminative value of all four indices was modest and became negligible after optimism correction. Exploratory analyses suggested possible sex-related heterogeneity for ASM/weight and CCR, whereas BMI-stratified findings were not supported by significant interaction tests. **Conclusions:** Estimated muscle-related indices may serve as potential risk markers in prediabetes, but their incremental predictive value beyond established risk factors is limited and requires external validation before clinical application.

## 1. Introduction

Prediabetes is an intermediate stage of glucose metabolism between normoglycemia and overt type 2 diabetes mellitus (T2DM), which affects about 35.2% of Chinese individuals [1,2]. The annual incidence of progression to T2DM in those with prediabetes is 5–10% and the cumulative rate of progression may be as high as 50% over 5–10 years [3]. Importantly, prediabetes is a potentially reversible condition, and timely identification of modifiable risk factors can facilitate targeted interventions to prevent or delay its progression to diabetes [4]. However, current risk stratification is primarily based on glycemic indicators alone, with insufficient ability to capture the complex metabolic pathways underpinning development. Therefore, there is an urgent need to identify attainable and biologically relevant markers to improve risk prediction in this high-risk population.

Skeletal muscle is essential for glucose metabolism and serves as a primary site for glucose uptake and storage [5]. Alterations in skeletal muscle mass are potentially linked to impaired glucose metabolism and may contribute to insulin resistance and abnormal glucose regulation [6,7]. Importantly, earlier research has indicated that individuals with prediabetes have less muscle mass and strength than healthy controls, and observational evidence suggests an association between sarcopenia and elevated diabetes risk [8,9]. These observations suggest that muscle-related indices may offer mechanistic insights into the transition from prediabetes to overt T2DM.

However, direct measurement of muscle mass in large epidemiological studies is challenging. Standard measurement techniques, including magnetic resonance imaging (MRI), dual-energy X-ray absorptiometry (DXA), computed tomography (CT), and bioelectrical impedance analysis (BIA), are costly and operationally complex, which limits their routine use in clinical and community settings [10]. In this study, we used four estimated muscle-related indices derived from routine anthropometric and biochemical data as surrogate measures. Among these, the height-adjusted appendicular skeletal muscle mass (ASM/height^2^) is currently a widely used diagnostic indicator for sarcopenia internationally and has been endorsed by the Asian Sarcopenia Working Group [11]. The body weight-adjusted ASM (ASM/weight) represents the ratio of muscle mass to total body weight, linking more direct reflection of the obesity phenotype [12]. The creatinine-to-cystatin C ratio (CCR) leverages the fact that creatinine originates from muscle metabolism while cystatin C does not, thus indirectly estimating muscle mass [13,14]. And the predicted skeletal muscle index (pSMI) further integrates CCR, hemoglobin, body weight, and age, and has been used as a composite surrogate measure of muscle mass in epidemiological studies [15,16].

Although an association between these indices and T2DM has been identified in earlier studies, the conclusions remain inconsistent due to different study populations, study designs, and measurement methods [17,18,19,20]. Moreover, most previous studies have focused on a single index in general populations, leaving an important gap: whether estimated muscle-related indices are associated with progression from prediabetes to T2DM, and whether they provide incremental discriminative value beyond conventional risk factors, remains unclear. Therefore, this study aimed to investigate the relationships of four estimated muscle-related indices—ASM/height^2^, ASM/weight, CCR, and pSMI—with progression from prediabetes to T2DM in a retrospective cohort with up to 10 years of follow-up. We further evaluated their incremental discriminative value using time-dependent AUC and ΔAUC, and explored whether these associations varied according to sex and BMI. By using routinely available anthropometric and biochemical variables, we sought to assess the potential utility of these estimated muscle-related indices as surrogate measures of muscle mass for risk stratification among individuals with prediabetes. These indices should currently be regarded as potential risk markers rather than validated tools for individualized risk assessment, and further validation in independent populations and prospective studies is needed.

## 2. Methods

### 2.1. Study Subjects

Our research enrolled adults who received physical examinations from 1 January 2015 to 31 December 2025, at the Second Affiliated Hospital of Zhejiang University School of Medicine. Inclusion criteria were (1) fulfilling the prediabetes diagnostic criteria [21]; (2) being 18 years of age or older; and (3) having at least one physical examination record during the follow-up period. The exclusion criteria included (1) a confirmed diabetes diagnosis at baseline (T2DM, gestational diabetes, or type 1 diabetes mellitus); (2) severe impairment of hepatic or renal function, characterized by an estimated glomerular filtration rate (eGFR) below 60 mL/min/1.73 m^2^ [22], alanine aminotransferase (ALT) > 120 U/L, or physician-diagnosed chronic hepatitis, cirrhosis, or nephritis; (3) a history of malignant tumors; (4) use of medications affecting muscle metabolism (e.g., glucocorticoids and androgens, etc.); (5) missing key variables; and (6) pregnant or lactating women. The screening process is shown in Supplementary Figure S1. The baseline date for each participant was defined as the date of the first physical examination from 1 January 2015 to 31 December 2015 at which the diagnostic criteria for prediabetes were first met. The primary outcome was the occurrence of incident T2DM during the follow-up period up to 31 December 2025. The follow-up duration was calculated from the baseline date to the date of the first medical examination at which the diagnostic criteria for T2DM were met. Because follow-up examinations were conducted at non-uniform intervals, the exact date of T2DM onset could not be determined with precision. Accordingly, the observed event date represents the first examination at which T2DM was identified, rather than the exact biological onset of diabetes. For participants who did not develop T2DM during the available observation period, the date of the last available physical examination was used as the censoring date.

### 2.2. Data Collection

Sex, age, lifestyle (current smoker [yes vs. no] and current drinker [yes vs. no]), and past medical history (hyperlipidemia, hypertension, malignant tumors, and diabetes) were obtained through interviews by trained healthcare professionals. Trained staff measured waist circumference (WC), height, and weight. The body mass index (BMI) was computed as weight (kg) divided by the square of height (m). Blood pressure was assessed by professional healthcare personnel with a digital sphygmomanometer (HBP-9020, Omron Co., Ltd., Tokyo, Japan). Blood samples (fasted > 8 h) were analyzed using a standardized automated biochemical analyzer (BECKMAN COULTER AU5800, Beckman Coulter, Inc., Brea, CA, USA). Biochemical parameters were uric acid (UA), albumin, alanine aminotransferase (ALT), hypersensitive C-reactive protein (hs-CRP), triglycerides (TGs), high-density lipoprotein cholesterol (HDL-C), fasting blood glucose (FBG), creatinine, hemoglobin, cystatin C, and glycated hemoglobin A1c (HbA1c). All tests were performed in the central laboratory of the Second Affiliated Hospital of Zhejiang University School of Medicine, which is certified to the ISO 15189 international standard [23].

### 2.3. Definition of Diseases

Prediabetes was defined based on the Chinese Diabetes Society guidelines [21]: HbA1c 5.7–6.4%, or FBG 6.1–6.9 mmol/L. T2DM was characterized as meeting any of the following criteria [21]: HbA1c ≥ 6.5%, FBG ≥ 7.0 mmol/L, current use of antidiabetic medication, or self-reported history of diabetes. Hypertension was defined as any of the following [24]: diastolic blood pressure (DBP) ≥ 90 mmHg, systolic blood pressure (SBP) ≥ 140 mmHg, current use of antihypertensive medication, or self-reported history of hypertension.

### 2.4. Calculation of Muscle Metrics

This study included four estimated muscle-related indices, all derived from routine anthropometric and biochemical data. ASM was calculated by applying an anthropometric equation that had been previously validated in Chinese adults [25]. In the original development sample, this model had an R^2^ of 0.90 and a standard error of estimate of 1.63 kg. In cross-validation, the mean difference between DXA-measured and estimated ASM was −0.025 kg (SD 1.63 kg), the paired *t*-test was non-significant (*p* = 0.761), and the correlation between measured and predicted ASM was r = 0.941. Male is designated a value of 1, whereas female is designated a value of 2:

ASM(kg) = 0.107 × height(cm) − 4.157 × gender + 0.193 × weight(kg) − 0.037 × age(year) − 2.631

Based on this, we calculated the following indices:

ASM/height^2^ (kg/m^2^) = ASM(kg)/height^2^(m)

ASM/weight = ASM(kg)/weight(kg)

CCR = Cr(mg/dL)/CysC(mg/L)

pSMI(male) = 4.17 + 1.24 × CCR + 0.0598 × weight(kg) − 0.0513 × Hb(g/dL) − 0.012 × age(year)

pSMI(female) = 3.55 + 0.852 × CCR + 0.0614 × weight(kg) − 0.0627 × Hb(g/dL) − 0.00765 × age(year)

### 2.5. Statistical Analysis

The distribution of continuous variables was examined through normality tests in conjunction with Q-Q plots. Continuous variables with normal distribution were represented as mean ± standard deviation (SD), and group comparisons were made using independent samples *t*-tests. Continuous variables with non-normal distribution were presented as medians (Q_1_, Q_3_) and compared by the Mann–Whitney U test. Categorical variables were expressed as frequencies and percentages, and group comparisons were made by the chi-square test or Fisher’s exact test. The pairwise correlations among the four estimated muscle-related indices were examined by Spearman’s rank correlation analysis. Because the prediction equations used to derive the estimated muscle-related indices were sex-specific, each index was categorized into sex-specific tertiles (low, medium, and high). The cumulative probability of T2DM progression was estimated by Kaplan–Meier curves, and the log-rank test was applied to evaluate differences between groups.

Cox proportional hazards regression models were performed to evaluate the associations between each estimated muscle-related index and progression from prediabetes to T2DM. Schoenfeld residuals were used to test the proportional hazards assumption, which revealed no significant violations (all *p* > 0.05). Each index was analyzed both as a continuous variable (per 1-SD increase) and according to sex-specific tertiles, with the lowest tertile serving as the reference category. Hazard ratios (HRs) and 95% confidence intervals (CIs) were estimated. Three sequential models were constructed: Model 1 was unadjusted; Model 2 was adjusted for sex; and Model 3 was further adjusted for smoking status, alcohol intake, hypertension, WC, FBG, TGs, HDL-C, hs-CRP, albumin, and ALT.

To evaluate potential multicollinearity, variance inflation factors (VIFs) were calculated for the covariates included in each multivariable model. All VIFs for the covariates in the prespecified Model 3 were below 5. Since age was already incorporated into the construction of the pSMI and ASM and is relevant to the derivation of the estimated muscle-related indices, it was excluded from the prespecified primary model to avoid potential construct overlap and the risk of overfitting. Baseline HbA1c was not included in the prespecified primary model because it is a direct marker of baseline glycemic status and was used, together with FBG, to define prediabetes at study entry. However, to evaluate the robustness of the findings to additional adjustment for age and baseline glycemic status, sensitivity analyses were performed by separately adding age, HbA1c, and age plus HbA1c to Model 3. The VIFs for age and the corresponding estimated muscle-related indices in these age-adjusted models are reported in Supplementary Table S1. Because the four estimated muscle-related indices were correlated to varying degrees, each index was entered separately into the Cox models rather than simultaneously. To account for multiple comparisons among the four primary index associations, false discovery rate (FDR) adjustment was performed using the Benjamini–Hochberg procedure.

Restricted cubic spline (RCS) analyses were carried out to investigate the potential nonlinear associations between each estimated muscle-related index and the risk of T2DM progression. Three knots were placed at the 10th, 50th, and 90th percentiles, with the median value used as the reference. Both the overall association and the departure from linearity were assessed using the corresponding Wald tests. The RCS models were fitted using the same covariate adjustment strategy as the Cox regression models. Time-dependent C-indices and time-dependent areas under the receiver operating characteristic curves (AUCs) were estimated at 1, 3, 5, 8, and 10 years of follow-up. A baseline model containing the clinical and biochemical covariates included in Model 3 was compared with models obtained by adding each estimated muscle-related index individually. The incremental discrimination provided by each index was assessed using the change in time-dependent AUC (ΔAUC) relative to the baseline model, with 95% CIs and formal statistical tests for the difference in AUCs. Because discrimination estimates may be optimistic when derived from the same cohort used for model estimation, internal validation was additionally performed using 1000 bootstrap resamples. Optimism-corrected ΔAUC values were calculated by subtracting the mean bootstrap optimism from the apparent ΔAUC, and percentile-based bootstrap 95% CIs were obtained. Harrell’s C-index, time-dependent AUCs, and ΔAUCs were calculated using the survival, timeROC, and riskRegression packages in R.

We performed subgroup analyzes for sex and BMI and investigated their interactions with estimated muscle-related indices. To evaluate the robustness of the primary findings, several sensitivity analyses were carried out. First, participants who developed T2DM within the first year of follow-up were excluded to reduce potential reverse causation and the influence of undiagnosed or evolving diabetes at baseline. Second, because the exact onset time of T2DM could not be directly observed and the event could only be ascertained between two examination visits, interval-censored survival analyses were performed as an additional sensitivity analysis. These analyses were conducted using Model 3 and the model additionally adjusted for age and HbA1c. A parametric log-logistic model was also fitted as an alternative specification of the interval-censored analysis. All statistical analyzes were performed using SPSS version 25.0 and R software version 4.4.3. All tests were two-sided. For the primary analyses, statistical significance was set at a *p* value < 0.05; for analyses involving multiple comparisons, FDR-adjusted *q* values were additionally considered.

### 2.6. Ethical Considerations

The Institutional Review Board of the Second Affiliated Hospital of Zhejiang University School of Medicine approved the study procedure on 1 April 2026 (Approval No: [<2026> Lunshenyan No. (0569)]). Given the retrospective design of the study and the use of anonymized data, the ethics committee waived the requirement for individual informed consent.

## 3. Results

### 3.1. Basic Information of the Study Objects

Among 927 individuals with prediabetes at baseline (696 males, 75.1%; 231 females, 24.9%), 211 cases (22.76%) progressed to new-onset T2DM during a median follow-up period of 4.23 years. The distribution of participants across prespecified follow-up horizons is summarized in Supplementary Table S2. At 1, 3, 5, 8, and 10 years, 841, 552, 439, 280, and 108 participants, respectively, remained at risk, while the corresponding cumulative numbers of T2DM events were 33, 114, 154, 189, and 204. The cumulative numbers of censored participants were 53, 261, 334, 458, and 615, respectively, corresponding to censoring rates of 5.72%, 28.16%, 36.03%, 49.41%, and 66.34%. The frequency of follow-up examinations also varied across participants. Among the 927 participants, 312 (33.66%) underwent one follow-up examination, 177 (19.09%) underwent two, 92 (9.92%) underwent three, 84 (9.06%) underwent four, and 262 (28.26%) underwent five or more follow-up examinations (Supplementary Table S3).

Table 1 displays baseline characteristics. Those who developed T2DM were older and had higher FBG, BMI, HbA1c, WC, ALT, TGs, and hs-CRP levels (*p* ≤ 0.001), as well as lower HDL-C levels, than those who did not (*p* = 0.001). Smoking and alcohol consumption were more common among participants who progressed to T2DM than among those who did not. Regarding estimated muscle-related indices, those who progressed to T2DM exhibited higher ASM/height^2^ and pSMI (*p* < 0.001).

### 3.2. Correlation Analysis of Estimated Muscle-Related Indices

Spearman’s rank correlation analysis (Supplementary Figure S2 and Table S4) revealed varying degrees of correlation among the four estimated muscle-related indices. ASM/height^2^ and pSMI had a significant positive connection (*r* = 0.883, *p* < 0.001). CCR and pSMI had a moderately favorable correlation (*r* = 0.508, *p* < 0.001), and the remaining indices showed low-to-moderate correlations with each other (*r* = 0.161–0.379, all *p* < 0.001).

### 3.3. Survival Analysis

The gender-specific tertile cutoffs of estimated muscle-related indices are provided in Supplementary Table S5. The results of unadjusted KM analysis and log-rank test showed that the high-score groups for pSMI (*p* = 0.029) and ASM/height^2^ (*p* < 0.001), as well as the low-score groups for CCR (*p* = 0.029) and ASM/weight (*p* < 0.001), showed the highest crude cumulative T2DM incidence (Supplementary Figure S3). Supplementary Table S6 lists the numbers at risk at each time point. It should be noted that the KM analysis was not adjusted for confounding factors. Therefore, the above results should be interpreted in conjunction with multivariate analysis.

### 3.4. Association Between Estimated Muscle-Related Indices and T2DM

Cox proportional hazards models were used to assess the associations between the four estimated muscle-related indices and incident T2DM (Table 2). In the primary multivariable-adjusted Model 3, each 1-SD increase in ASM/weight was inversely associated with incident T2DM (HR = 0.54 [0.34, 0.86], *p* = 0.009), whereas pSMI showed a weaker inverse association (HR = 0.73 [0.53, 0.99], *p* = 0.041). No statistically significant associations were observed for ASM/height^2^ (HR = 0.91 [0.60, 1.37], *p* = 0.639) or CCR (HR = 0.90 [0.78, 1.04], *p* = 0.155) (Supplementary Figure S4A).

When the indices were categorized into sex-specific tertiles, the highest tertile of ASM/weight was associated with a lower hazard of incident T2DM compared with the lowest tertile (HR = 0.56 [0.36, 0.89], *p* = 0.013), with a nominally significant trend across tertiles (*p* for trend = 0.012). The corresponding tertile associations for ASM/height^2^, CCR, and pSMI were not statistically significant (Supplementary Figure S4B).

To account for multiple testing across the four primary index associations, the Benjamini–Hochberg FDR procedure was applied. The FDR-adjusted *q*-values were 0.639 for ASM/height^2^, 0.036 for ASM/weight, 0.207 for CCR, and 0.082 for pSMI. Thus, the inverse association between ASM/weight and incident T2DM remained statistically significant after FDR adjustment, whereas the association for pSMI did not (Supplementary Table S7).

To examine the potential influence of age and baseline glycemic status on these associations, additional models were fitted by separately adding age, HbA1c, or both to Model 3. The associations were attenuated after additional adjustment for age and/or HbA1c. In the model additionally adjusted for both age and HbA1c, none of the four indices remained statistically significantly associated with incident T2DM (Supplementary Table S8). These analyses indicate that the associations observed in the primary Model 3 were sensitive to additional adjustment for age and baseline HbA1c.

### 3.5. Dose–Response Analysis

**Figure 1:**
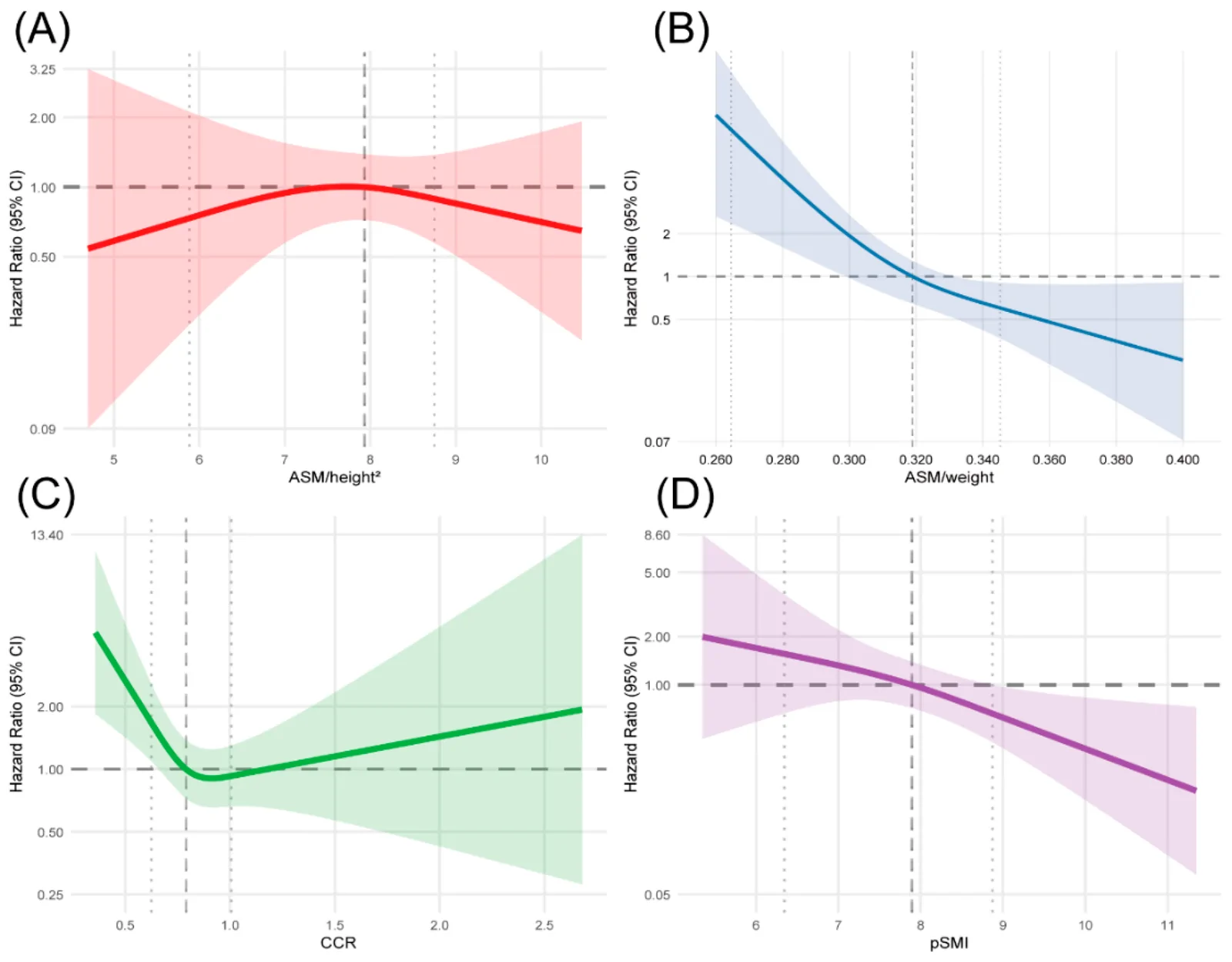
Dose-responsive relationship of estimated muscle-related indices with the risk of T2DM. (**A**) ASM/height^2^, (**B**) ASM/weight, (**C**) CCR, (**D**) pSMI. The model was adjusted for sex, smoking, drinking, hypertension, WC, TGs, HDL-C, ALT, hs-CRP, FBG and albumin. Adjusted HRs are depicted by solid lines, while shaded areas denote the 95% CIs. The horizontal dashed line indicates the reference hazard ratio of 1.0. The vertical dashed lines indicate the 10th, 50th, and 90th percentiles of the corresponding index, which were used as knots in the restricted cubic spline analysis.

RCS analyses were carried out to assess potential nonlinear associations between the estimated muscle-related indices and incident T2DM (Figure 1). In the primary Model 3, ASM/weight showed evidence of a nonlinear association with incident T2DM (*p*_total_ = 0.0001, *p*_nonlinear_ = 0.0215), whereas CCR demonstrated a nonlinear, U-shaped association (*p*_total_ = 0.0012, *p*_nonlinear_ = 0.0023). pSMI showed an overall inverse association without evidence of significant nonlinearity, while ASM/height^2^ showed no statistically significant overall or nonlinear association.

The evidence for nonlinearity was sensitive to additional covariate adjustment. For ASM/weight, the evidence of nonlinearity was attenuated after additional adjustment for age and was no longer statistically significant after adjustment for HbA1c or for both age and HbA1c. For CCR, evidence of nonlinearity was not observed after adjustment for age alone but remained nominally significant after additional adjustment for HbA1c or for both age and HbA1c (*p*_nonlinear_ = 0.0332 and 0.0441, respectively) (Supplementary Table S9).

### 3.6. Incremental Discriminative Value of Estimated Muscle-Related Indices

**Figure 2:**
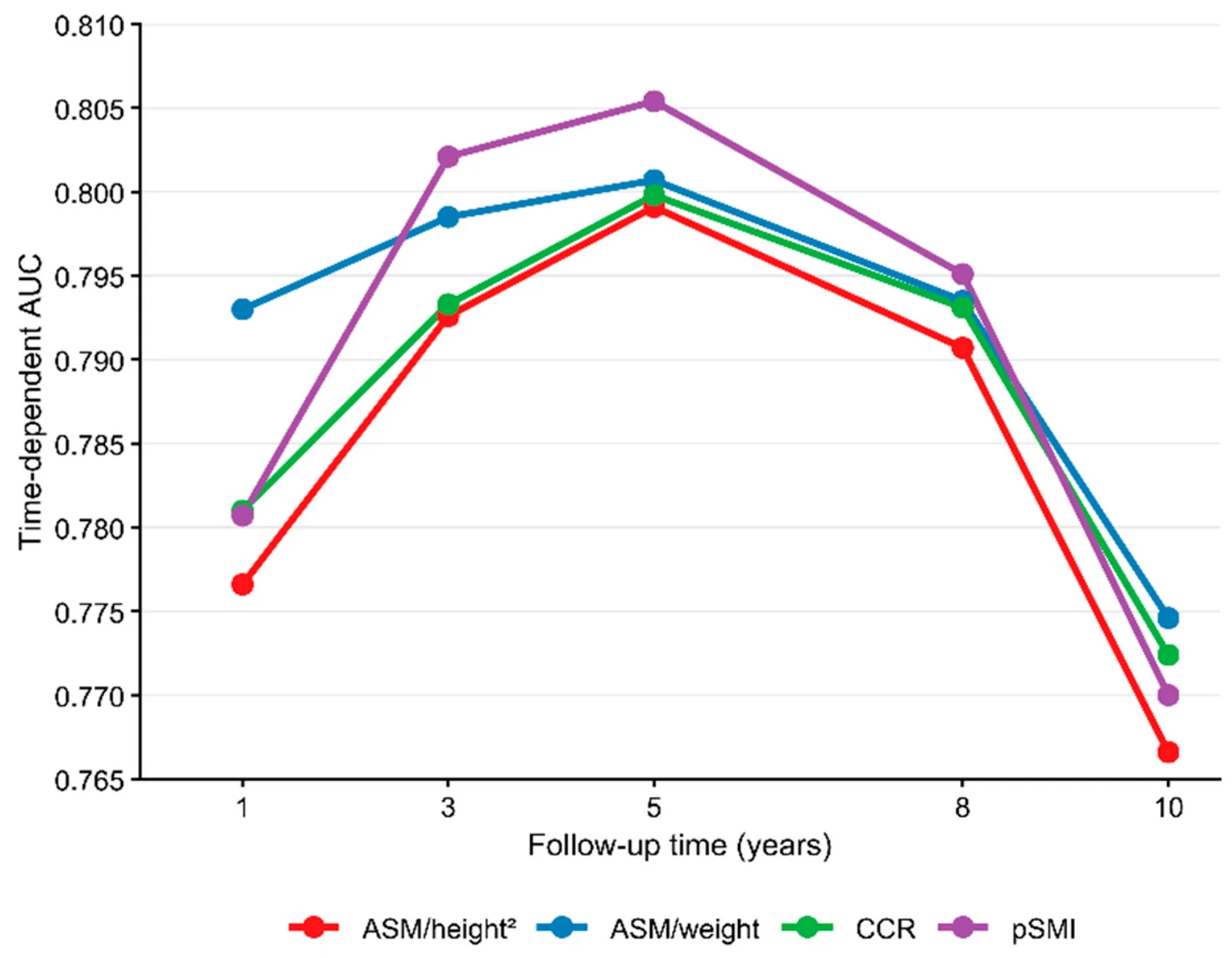
Time-dependent predictive capacity of estimated muscle-related indices for T2DM.

The discriminative performance of the prediction models was evaluated using Harrell’s C-index and time-dependent AUCs at 1, 3, 5, 8, and 10 years. Under the prespecified Model 3, the Harrell’s C-index was 0.7673 for the baseline model and 0.7677, 0.7725, 0.7684, and 0.7719 after adding ASM/height^2^, ASM/weight, CCR, and pSMI, respectively, indicating only modest differences in overall discrimination (Supplementary Table S10). Time-dependent AUCs across the prespecified follow-up horizons showed similarly small differences among the index-augmented models (Figure 2 and Supplementary Table S11).

Incremental discrimination was further assessed by formally comparing the time-dependent AUCs of each index-augmented model with those of the corresponding baseline model. Under Model 3, the ΔAUCs were generally small and not statistically significant. pSMI showed nominally significant incremental discrimination at 3 years (ΔAUC = 0.0103 [0.0007, 0.0199], *p* = 0.0351) and 5 years (ΔAUC = 0.0081 [0.0003, 0.0160], *p* = 0.0431), while the other indices and the remaining time points showed no statistically significant improvement in ΔAUC (Supplementary Table S12).

Bootstrap internal validation (1000 resamples) resulted in generally small optimism-corrected ΔAUCs. For pSMI, the optimism-corrected ΔAUC was 0.009 (95% CI: 0.0001–0.0280, *p* = 0.048) at 3 years and 0.007 (95% CI: −0.0002–0.022, *p* = 0.066) at 5 years; the corresponding estimates at 1, 8, and 10 years were not statistically significant. The other indices showed no consistent evidence of incremental discrimination after optimism correction (Supplementary Table S13 and Figure S5).

When age and baseline HbA1c were additionally incorporated into the baseline model, none of the four estimated muscle-related indices provided statistically significant incremental discrimination at 1, 3, 5, 8, or 10 years. The corresponding ΔAUC estimates were close to zero, and bootstrap optimism-corrected analyses likewise showed no consistent evidence of incremental discrimination (Supplementary Tables S12 and S13).

Calibration was additionally evaluated at 3, 5, and 8 years for the baseline Model 3 and the model additionally adjusted for age and baseline HbA1c. Calibration plots for the baseline model and each index-augmented model are presented in Supplementary Figures S6–S11. These analyses were performed separately from discrimination analyses to assess the agreement between predicted and observed risks across the prespecified time horizons.

### 3.7. Subgroup Analysis and Interaction Tests

#### 3.7.1. Sex Subgroup

Formal interaction testing indicated evidence of sex heterogeneity for ASM/weight (interaction HR = 0.19 [0.07, 0.52], *p* = 0.001) and CCR (interaction HR = 0.35 [0.20, 0.61], *p* < 0.001), whereas no significant interaction was observed for ASM/height^2^ (interaction HR = 1.38 [0.72, 2.65], *p* = 0.327) or pSMI (interaction HR = 0.94 [0.49, 1.78], *p* = 0.843) (Supplementary Table S14). In sex-stratified analyses, ASM/weight was inversely associated with incident T2DM in females (HR = 0.11 [0.03, 0.38], *p* < 0.001), whereas the association was weaker and did not reach statistical significance in males (HR = 0.62 [0.38, 1.02], *p* = 0.060). Similarly, CCR was inversely associated with incident T2DM in females (HR = 0.31 [0.17, 0.59], *p* < 0.001), but not in males (HR = 0.96 [0.84, 1.10], *p* = 0.558) (Supplementary Table S15).

In sensitivity analyses additionally adjusting for age and baseline HbA1c, the interaction patterns for ASM/weight and CCR were broadly similar, although the interaction estimate for ASM/weight was attenuated (Supplementary Table S14).

#### 3.7.2. BMI Subgroup

This study classified participants into normal weight (18.5–23.9 kg/m^2^, *n* = 367), overweight (24.0–27.9 kg/m^2^, *n* = 434), and obese groups (≥ 28.0 kg/m^2^, *n* = 109) [26]. Due to the absence of T2DM events in the underweight group (BMI < 18.5 kg/m^2^, *n* = 17), this group was merged into the normal-weight group, resulting in a final normal-weight group of 384 participants.

For BMI-stratified analyses, none of the four estimated muscle-related indices showed statistically significant interaction with BMI category (all interaction *p* > 0.05; Supplementary Table S16). Within individual BMI strata, ASM/weight was inversely associated with incident T2DM among normal-weight participants (HR = 0.22 [0.09, 0.56], *p* = 0.002) and overweight participants (HR = 0.41 [0.17, 0.96], *p* = 0.041), while pSMI was inversely associated with incident T2DM among obese participants (HR = 0.48 [0.24, 0.96], *p* = 0.039) (Supplementary Table S17).

### 3.8. Sensitivity Analyses

#### 3.8.1. Sensitivity Analysis Excluding Early Incident T2DM Cases

To assess the potential influence of reverse causation, we repeated the analyses after excluding 33 participants who developed T2DM within the first year of follow-up, leaving 894 participants and 178 T2DM events for the sensitivity analysis. The inverse association between pSMI and incident T2DM remained statistically significant (HR = 0.71 [0.51, 0.99], *p* = 0.049), although the magnitude was modest. ASM/height^2^ and CCR remained not significantly associated with incident T2DM. For ASM/weight, the association was attenuated and no longer reached conventional statistical significance (HR = 0.61 [0.37, 1.00], *p* = 0.051), although the direction of association remained unchanged (Supplementary Table S18).

#### 3.8.2. Sensitivity Analysis Accounting for Interval Censoring

Because T2DM onset could not necessarily be determined between two consecutive examinations, we performed an interval-censored survival analysis using the last examination without T2DM and the first examination meeting the T2DM diagnostic criteria to define the event interval. Under the primary Model 3 adjustment, the interval-censored analysis yielded estimates broadly consistent with those from the primary Cox analysis. For ASM/weight, the HR was 0.54 (95% CI: 0.34–0.86, *p* = 0.009) in the log-logistic model, compared with 0.54 (95% CI: 0.34–0.86, *p* = 0.010) in the corresponding Cox analysis. The associations for ASM/height^2^, CCR, and pSMI were also generally comparable between the two approaches. After additional adjustment for age and baseline HbA1c, the associations for all four estimated muscle-related indices were attenuated and no longer statistically significant (Supplementary Table S19).

## 4. Discussion

This study exploded the associations of four estimated muscle-related indices with the development of T2DM among adults with prediabetes and evaluated their incremental discriminative value in a retrospective cohort with up to 10 years of follow-up (median, 4.23 years). In the prespecified multivariable model, higher ASM/weight and pSMI were inversely associated with incident T2DM, whereas ASM/height^2^ and CCR were not. It should also be noted that the pSMI association was sensitive to the modelling strategy: although pSMI was statistically significant when modelled as a continuous variable, the tertile-based analysis was not statistically significant. After FDR correction, only the association for ASM/weight remained statistically significant. These associations were attenuated after additional adjustment for age and baseline HbA1c, and none of the four indices remained statistically significant after simultaneous adjustment for both. Despite these associations, the four indices provided only modest incremental discrimination beyond conventional risk factors, with no consistent improvement in ΔAUC after bootstrap optimism correction. A U-shaped association was observed for CCR, with lower estimated risk within the observed range of approximately 0.79–1.20, although this pattern was attenuated after additional adjustment. Exploratory subgroup analyses suggested possible sex-related heterogeneity for ASM/weight and CCR, whereas BMI-stratified findings were not supported by significant interaction tests. Overall, these findings indicated that estimated muscle-related indices were associated with progression from prediabetes to T2DM in the prespecified primary model, but these associations were not robust to multiple-testing correction, reverse-causation analysis, or additional adjustment for age and HbA1c. These indices should therefore be regarded as exploratory risk markers rather than established predictors of T2DM progression, although they may still help identify individuals with prediabetes who warrant further risk assessment.

Wan et al. reported that a higher ASM/weight ratio was significantly associated with a reduced risk of progression to diabetes and a greater likelihood of reverting to normal blood glucose levels among middle-aged and older Chinese adults with prediabetes [12]. Similarly, a large-scale cohort study involving 47,885 individuals reported that among women, an elevated pSMI was related to a decreased risk of diabetes [27]. These findings are generally consistent with the inverse associations observed for ASM/weight and pSMI in our study and provide further support for a relationship between estimated muscle-related indices and subsequent diabetes risk. However, the results were not uniform across different muscle-related indices. Wang et al. reported a modest, but statistically nonsignificant, inverse association between CCR and incident diabetes [20], which is more comparable to our findings. Zhang et al. showed that a lower ASM/height^2^ was linked to an increased risk of incident diabetes and supported a bidirectional longitudinal relationship between sarcopenia and diabetes [19], whereas ASM/height^2^ showed no significant association with T2DM in our study. These differences may partly reflect variation in study populations, definitions and estimation methods for muscle-related measures, outcome definitions, and covariate adjustment. The possibility of a bidirectional relationship is particularly relevant to our findings. When participants who developed T2DM within the first year of follow-up were excluded in the sensitivity analysis, the inverse association for ASM/weight was weakened and no longer reached statistical significance. The specific mechanism is that skeletal muscle constitutes the principal site of insulin-mediated glucose uptake, and then reduced muscle mass impairs glucose homeostasis. More importantly, a bidirectional vicious cycle exists between muscle and insulin resistance. Insulin resistance aggravates muscle catabolism, while a further reduction in muscle mass, in turn, worsens insulin resistance [28].

The substantial change in the ASM/weight estimate after adjustment for sex alone (Model 1: HR = 0.98, Model 2: HR = 0.41) is explained by the fact that the anthropometric equation used to estimate ASM is sex-specific, so ASM/weight is strongly correlated with sex. In our cohort, males had higher ASM/weight values and a higher incidence of T2DM than females, and sex therefore acted as a strong confounder of the crude association. We also found that ASM/height^2^ and pSMI exhibited opposite directions of effect across different adjustment models. In the KM analysis and Models 1 and 2, higher muscle indices were linked with an increased risk of T2DM. However, after further adjustment for WC and other metabolic risk factors, both indices showed an inverse direction of association. The underlying reason for this phenomenon is that body weight, which is incorporated into the calculation of these indices, reflects both muscle and fat mass. While higher weight may indicate greater muscle mass, it often coexists with excess adiposity, particularly visceral fat, which is a well-established driver of insulin resistance and T2DM [29]. This issue is particularly relevant to ASM/weight, for which body weight is incorporated into both the estimated ASM equation and the index denominator. The strong inverse association observed for ASM/weight may therefore partly arise from its mathematical relationship with body weight and adiposity, rather than solely from an underlying muscle-specific effect. Consistent with this, ASM/weight was nearly identical between participants who did and did not develop T2DM in the unadjusted baseline comparison, yet it showed the strongest adjusted association with T2DM among the four indices. This apparent discrepancy reflects the fact that the baseline comparison is unadjusted and does not account for sex or body weight. Accordingly, ASM/weight should not be interpreted as an independent index of muscle biology. In exploratory analyses, the inverse association of ASM/weight was observed in normal-weight and overweight participants but not in obese participants, suggesting that above a certain adiposity threshold, the metabolic consequences of excess fat may outweigh the beneficial effects of muscle mass [30,31]. These BMI-stratified findings should nevertheless be interpreted cautiously because the formal interaction tests were not statistically significant and the obese subgroup was small. Mechanistically, skeletal muscle and adipose tissue communicate through myokines and adipokines, and excess adiposity may impair muscle insulin signaling and glucose handling [32,33].

In our cohort, CCR showed a U-shaped, nonlinear association with incident T2DM. In the primary Model 3, the risk of T2DM was higher when CCR was below approximately 0.79 or above approximately 1.20. This finding differs from the linear negative correlation reported in most previous studies [20,34]. This may be because the subjects in this study were in the prediabetic stage, which is a phase with metabolic dysfunction and potentially more complex pathophysiological mechanisms. Han et al. reported that a decreased CCR reflects low muscle mass and strength, leading to impaired glucose handling capacity [35], and Osaka et al. recommend a CCR of 0.9 as the optimal threshold for identifying sarcopenia in patients with T2DM [14]. However, CCR is also influenced by factors other than muscle, including renal function, hydration status, creatinine metabolism, and dietary protein intake [36]. An excessively high CCR may therefore reflect conditions such as dehydration, high protein intake, early renal dysfunction, or increased muscle catabolism, rather than a beneficial muscle phenotype [37].

In this study, sex is another key factor modulating the association between estimated muscle-related indices and diabetes risk, consistent with previous studies [27]. In Model 3, formal interaction tests were significant for ASM/weight and CCR. After additional adjustment for age and HbA1c, the interaction remained significant. These estimates indicate that the inverse associations of ASM/weight and CCR with incident T2DM were stronger in females than in males. The sex-stratified estimates are consistent with this pattern. Possible mechanisms include estrogen-related effects on muscle insulin sensitivity [38], and sex differences in muscle fiber composition [39,40] and fat distribution [41,42]. However, only 33 female participants developed T2DM in our study, and the confidence intervals for the female-specific estimates were wide. The interaction estimates themselves should therefore also be interpreted with caution.

The incremental discriminative value of the four estimated muscle-related indices was modest and largely attenuated after adjustment for age and HbA1c. Several explanations may account for the limited incremental discriminative value observed. First, the four indices are derived from routine anthropometric and biochemical variables that overlap substantially with components already included in the baseline model. For instance, pSMI includes age, weight, hemoglobin, and CCR, many of which are themselves associated with T2DM risk. Consequently, the information carried by these indices may be largely redundant with that already captured by established risk factors, leaving limited room for incremental improvement. Second, the modest incremental value may reflect the inherent limitations of the indices as surrogate measures. ASM was estimated using an anthropometric equation rather than directly measured by DXA, BIA, CT, or MRI. This estimation approach may introduce measurement error and reduce the ability of these indices to capture true muscle-related risk beyond what is already reflected in conventional risk factors. Similarly, CCR is influenced by renal function, hydration status, and protein intake, and thus may not specifically reflect muscle mass in all individuals. Third, the absence of incremental value after adjustment for age and HbA1c is particularly informative. Both age and HbA1c are strong predictors of T2DM progression, and the estimated muscle-related indices may partly capture information already conveyed by these variables. This is consistent with the VIF results demonstrating substantial collinearity between ASM-based indices and age (VIF > 8), and with the attenuation of associations after age and HbA1c adjustment. Currently, a large number of observational studies have confirmed an association between muscle-related indicators and the onset and progression of diabetes [17]. Our findings extend these observations by showing that, despite significant associations, the incremental discriminative contribution of estimated muscle-related indices is limited.

From a clinical perspective, the estimated muscle-related indices used in this study are derived from routine anthropometric and biochemical data and may therefore be easily obtainable in primary care and health examination settings, where they could help identify individuals with prediabetes who are at higher risk of progression to T2DM. However, these indices may serve primarily as risk markers that reflect underlying metabolic and body composition states, rather than as independent predictors that meaningfully enhance risk stratification. Future studies should therefore prioritize external validation in independent, multicenter, and multiethnic cohorts to confirm whether these indices provide incremental predictive value beyond conventional risk factors. In addition, repeated assessments of muscle-related indices over time, or combinations of these indices with direct measures of muscle function such as handgrip strength and gait speed, may better capture the dynamic and multifaceted nature of muscle health in relation to T2DM risk. Decision curve analysis should also be conducted to assess whether the use of these indices in clinical decision-making yields net benefit across clinically relevant risk thresholds. Given that CCR is influenced by dietary protein intake, hydration status, and renal function, future studies with comprehensive assessment of these factors are needed to clarify its biological determinants. Ultimately, randomized controlled trials will be required to determine whether screening strategies incorporating estimated muscle-related indices can contribute to the prevention of T2DM in high-risk populations.

The strengths of this study include the follow-up of up to 10 years, the comparison of four estimated muscle-related indices within the same cohort, the use of RCS analysis to flexibly model nonlinear associations, and the systematic evaluation of incremental discriminative value through time-dependent AUC, formal ΔAUC comparisons, bootstrap internal validation, and calibration. These features improve the methodological rigor and clinical relevance of the findings. However, there are certain limitations. First, this study was a single-center cohort study in East China, and the generalizability of the conclusions to populations in other areas needs to be further validated. Second, muscle indices were computed by the anthropometric equation. Although this equation was developed and cross-validated in Chinese adults with satisfactory performance (R^2^ = 0.90, SEE = 1.63 kg), it was not specifically validated in a prediabetic population or in the age and sex distribution of the present cohort, and non-differential measurement error may therefore have attenuated the observed associations. Third, prediabetes was defined using HbA1c and FBG only, and OGTT was not available in this physical examination cohort. Therefore, individuals with isolated impaired glucose tolerance may have been missed. Fourth, the date of the initial physical examination that satisfied diagnostic requirements was designated as the T2DM diagnosis date, rather than the actual onset date, which could have happened between examinations. Although interval-censored analyses were performed, residual misclassification of event timing may remain. Fifth, follow-up examinations were conducted at non-uniform intervals, and the frequency of surveillance varied across participants, which may have influenced the timing of event detection and introduced residual bias. Sixth, because this was a retrospective physical examination cohort, reasons for loss to follow-up were not systematically recorded and could not be retrieved. Seventh, physical activity, dietary intake, participation in diabetes-prevention or weight-loss interventions, and subsequent medication use were not available in this retrospective cohort. These factors could substantially influence progression from prediabetes to T2DM and may also be associated with the estimated muscle-related indices, and the direction and magnitude of the resulting residual confounding are difficult to predict. For example, individuals with higher physical activity and healthier dietary patterns may have both more favorable muscle-related indices and a lower risk of T2DM progression, which could inflate the apparent inverse associations observed in this study. Conversely, participants at higher metabolic risk may be more likely to receive lifestyle counselling or preventive interventions during follow-up, which could attenuate the observed associations. The absence of information on subsequent antidiabetic or weight-loss medication use is particularly relevant, because treatment initiation during follow-up could alter both the timing of T2DM detection and the trajectory of the muscle-related indices. Eighth, all muscle-related indices in this investigation were based on a single baseline assessment, and so the study was unable to analyze the dynamic influence of fluctuations in muscle mass over time on the course of diabetes. Finally, external validation and decision curve analysis were not performed, and the net clinical benefit of these indices remains uncertain.

## 5. Conclusions

In this retrospective cohort of Chinese adults with prediabetes, four estimated muscle-related indices derived from routine data showed different patterns of association with progression to T2DM. Higher ASM/weight and pSMI were inversely associated with incident T2DM in the prespecified primary model, but only ASM/weight remained significant after FDR correction, and all associations were attenuated after additional adjustment for age and HbA1c. CCR showed a U-shaped association, with an observed lower-risk range of approximately 0.79–1.20 that should not be interpreted as a clinical target. Despite these associations, the incremental discriminative value of all four indices was modest and became negligible after bootstrap optimism correction. Taken together, the most defensible finding of this study is that estimated muscle-related indices showed only limited and non-robust associations with progression from prediabetes to T2DM. They should therefore be regarded as exploratory risk markers rather than established predictors, and external validation in independent, multicenter, and multiethnic cohorts is needed before they can be considered for individualized risk assessment in clinical practice.

## Figures and Tables

**Table 1:** Basic characteristics of study participants.

Variables	Overall	Subsequent T2DM (*n* = 211)	Subsequent Non-T2DM (*n* = 716)	*p*
Age (years)	50.00 (43.00, 55.00)	52.00 (46.00, 56.00)	49.00 (42.25, 54.00)	<0.001
Sex				<0.001
Male	696 (75.1)	178 (84.4)	518 (72.3)	
Female	231 (24.9)	33 (15.6)	198 (27.7)	
BMI (kg/m^2^)	24.68 (22.29, 26.45)	25.39 (23.60, 27.09)	24.35 (22.10, 26.23)	<0.001
WC (cm)	86.47 ± 8.96	89.25 ± 7.44	85.66 ± 9.20	<0.001
Smoking (%)				<0.001
No	483 (52.1)	86 (40.8)	397 (55.4)	
Yes	444 (47.9)	125 (59.2)	319 (44.6)	
Drinking (%)				0.002
No	443 (47.8)	81 (38.4)	362 (50.6)	
Yes	484 (52.2)	130 (61.6)	354 (49.4)	
Hypertension (%)				0.001
No	583 (62.9)	113 (53.6)	470 (65.6)	
Yes	344 (37.1)	98 (46.4)	246 (34.4)	
TGs (mmol/L)	1.46 (1.03, 2.13)	1.69 (1.22, 2.47)	1.36 (0.97, 2.04)	<0.001
HDL-C (mmol/L)	1.13 (0.96, 1.33)	1.07 (0.92, 1.25)	1.14 (0.97, 1.35)	0.001
FBG (mmol/L)	5.12 (4.81, 5.53)	5.58 (5.14, 6.07)	5.05 (4.75, 5.35)	<0.001
ALT (U/L)	22.00 (16.00, 31.00)	25.00 (19.00, 36.00)	21.00 (15.00, 30.00)	<0.001
hs-CRP (mg/L)	0.80 (0.50, 1.80)	1.00 (0.60, 1.90)	0.80 (0.40, 1.70)	0.006
Albumin (g/L)	43.70 (42.00, 45.20)	43.50 (41.80, 45.10)	43.70 (42.00, 45.30)	0.361
HbA1c (%)	5.80 (5.70, 6.00)	6.10 (5.90, 6.20)	5.80 (5.70, 5.90)	<0.001
ASM/height^2^ (kg/m^2^)	7.93 (7.01, 8.36)	8.14 (7.58, 8.50)	7.86 (6.76, 8.31)	<0.001
ASM/weight	0.32 (0.30, 0.33)	0.32 (0.31, 0.33)	0.32 (0.29, 0.33)	0.688
CCR	0.79 (0.70, 0.90)	0.81 (0.68, 0.91)	0.79 (0.71, 0.90)	0.803
pSMI	7.89 (7.12, 8.42)	8.11 (7.52, 8.60)	7.83 (7.00, 8.34)	<0.001

T2DM, type 2 diabetes mellitus; BMI, body mass index; WC, waist circumference; TGs, triglyceride; HDL-C, high-density lipoprotein cholesterol; FBG, fasting blood glucose; ALT, alanine aminotransferase; hs-CRP, hypersensitive C-reactive protein; HbA1c, glycated hemoglobin A1c; ASM, appendicular skeletal muscle mass; CCR, Creatinine/Cystatin C Ratio; pSMI, predicted skeletal muscle mass index.

**Table 2:** Multivariable Cox regression models for T2DM.

	ASM/Height^2^		ASM/Weight		CCR		pSMI	
	HR (95% CI)	*p*	HR (95% CI)	*p*	HR (95% CI)	*p*	HR (95% CI)	*p*
Model 1								
Per 1-SD increase	1.47 (1.26–1.71)	<0.001	0.98 (0.86–1.12)	0.746	0.95 (0.82–1.10)	0.453	1.30 (1.13–1.49)	<0.001
Tertile 1 (reference)	1	-	1	-	1	-	1	-
Tertile 2	1.46 (1.02–2.09)	0.037	0.59 (0.44–0.81)	0.001	0.70 (0.50–0.98)	0.036	1.16 (0.82–1.64)	0.402
Tertile 3	2.04 (1.45–2.87)	<0.001	0.38 (0.27–0.54)	<0.001	0.68 (0.49–0.94)	0.018	1.54 (1.11–2.15)	0.011
Model 2								
Per 1-SD increase	1.60 (1.26–2.02)	<0.001	0.41 (0.31–0.55)	<0.001	0.83 (0.70–0.98)	0.025	1.20 (0.99–1.45)	0.056
Tertile 1 (reference)	1	-	1	-	1	-	1	-
Tertile 2	1.46 (1.02–2.09)	0.039	0.60 (0.44–0.81)	0.001	0.71 (0.51–1.00)	0.047	1.15 (0.81–1.63)	0.434
Tertile 3	2.03 (1.45–2.86)	<0.001	0.38 (0.27–0.54)	<0.001	0.68 (0.49–0.94)	0.018	1.54 (1.10–2.14)	0.011
Model 3								
Per 1-SD increase	0.91 (0.60–1.37)	0.639	0.54 (0.34–0.86)	0.009	0.90 (0.78–1.04)	0.155	0.73 (0.53–0.99)	0.041
Tertile 1 (reference)	1	-	1	-	1	-	1	-
Tertile 2	1.03 (0.70–1.51)	0.887	0.74 (0.52–1.06)	0.100	0.81 (0.58–1.13)	0.218	0.79 (0.53–1.15)	0.219
Tertile 3	1.16 (0.73–1.84)	0.533	0.56 (0.36–0.89)	0.013	0.79 (0.57–1.09)	0.153	0.96 (0.61–1.50)	0.850
*p* value for trend		0.527		0.012		0.147		0.944

Tertile 1: Low group; Tertile 2: Medium group; Tertile 3: High group. Model 1: Unadjusted. Model 2: Adjusted for sex. Model 3: Adjusted for sex, smoking, drinking, hypertension, WC, FBG, TGs, HDL-C, ALT, hs-CRP, and albumin.

## Data Availability

The original contributions presented in this study are included in the article/Supplementary Materials. Further inquiries can be directed to the corresponding author.

## Supplementary Materials

The following supporting information can be downloaded at https://www.mdpi.com/article/10.3390/jcm15207828/s1, Table S1. VIF for estimated muscle-related indices and age in age-adjusted models. Table S2. Numbers at risk, cumulative T2DM events, and censoring during follow-up. Table S3. Distribution of follow-up examination frequency. Table S4. Results of the correlation analysis of estimated muscle-related indices. Table S5. Tertile ranges of estimated muscle-related indices stratified by sex. Table S6. Number of participants at risk for Kaplan-Meier survival analysis at specified follow-up time points. Table S7. FDR-adjusted *p* values for the primary associations. Table S8. Associations of estimated muscle-related indices with incident T2DM after additional adjustment for age and/or HbA1c. Table S9. RCS analyses of estimated muscle-related indices and incident T2DM. Table S10. Harrell’s C-index of the baseline model and models with each estimated muscle-related index added. Table S11. Time-dependent AUCs of the prediction models at prespecified follow-up horizons. Table S12. Incremental discrimination of the estimated muscle-related indices assessed by time-dependent ΔAUC. Table S13. Bootstrap optimism-corrected incremental discrimination of the estimated muscle-related indices. Table S14. Sex interaction analyses between estimated muscle-related indices and incident T2DM. Table S15. Sex subgroup analysis of the association between estimated muscle-related indices and the risk of incident T2DM. Table S16. BMI interaction analyses between estimated muscle-related indices and incident T2DM. Table S17. BMI subgroup analysis of the association between estimated muscle-related indices and the risk of incident T2DM. Table S18. Sensitivity analysis results of the association between estimated muscle-related indices and the risk of incident T2DM (excluding early incident T2DM cases). Table S19. Sensitivity analysis using interval-censored survival models. Figure S1. Flowchart of the inclusion process of the study. Figure S2. Spearman’s correlation of estimated muscle-related indices. Figure S3. Kaplan-Meier curves for cumulative incidence of T2DM by the tertile groups of estimated muscle-related indices. (A) ASM/height^2^; (B) ASM/weight; (C) CCR; (D) pSMI. (A) ASM/height^2^; (B) ASM/weight; (C) CCR; (D) pSMI. Figure S4. Cox regression forest plot. (A) Continuous variables. (B) Categorical variable. Figure S5. Bootstrap optimism-corrected time-dependent AUCs of the prediction models. Figure S6 Calibration plots of the prediction model (Model 3) at 3 years. Figure S7 Calibration plots of the prediction model (Model 3) at 5 years. Figure S8. Calibration plots of the prediction model (Model 3) at 8 years. Figure S9. Calibration plots of the prediction model (Model 3 + Age + HbA1c) at 3 years. Figure S10. Calibration plots of the prediction model (Model 3 + Age + HbA1c) at 5 years. Figure S11. Calibration plots of the prediction model (Model 3 + Age + HbA1c) at 8 years.

## Abbreviations

The following abbreviations are used in this manuscript:

T2DM	Type 2 diabetes mellitus
ASM	Appendicular skeletal muscle mass
CCR	Creatinine-to-cystatin C ratio
pSMI	Predicted skeletal muscle mass index
DXA	Dual-energy X-ray absorptiometry
CT	Computed tomography
MRI	Magnetic resonance imaging
BIA	Bioelectrical impedance analysis
eGFR	Estimated glomerular filtration rate
WC	Waist circumference
BMI	Body mass index
ALT	Alanine aminotransferase
UA	Uric acid
hs-CRP	Hypersensitive C-reactive protein
TGs	Triglycerides
HDL-C	High-density lipoprotein cholesterol
FBG	Fasting blood glucose
HbA1c	Glycated hemoglobin A1c
SBP	Systolic blood pressure
DBP	Diastolic blood pressure
SD	Standard deviation
HRs	Hazard ratios
CIs	Confidence intervals
VIF	Variance inflation factor
KM	Kaplan–Meier
RCS	Restricted cubic spline
FDR	False discovery rate
