# Supplementary material for: Association and Incremental Discriminative Value of Four Estimated Muscle-Related Indices for Progression from Prediabetes to Type 2 Diabetes Mellitus: A Retrospective Cohort Study with up to 10 Years of Follow-Up
Source: J Clin Med. 2026 Oct 10;15(20):7828. doi: 10.3390/jcm15207828 (PMC13705024; doi:10.3390/jcm15207828)
Supplement: Supplementary file 1 [file jcm-15-07828-s001.zip › jcm-4550022-supplementary.pdf]

Association and Incremental Discriminative Value of Four Estimated Muscle-Related Indices for Progression from Prediabetes to Type 2 Diabetes Mellitus: A Retrospective Cohort Study with up to 10 Years of Follow-up

Appendix 1. Study population and follow-up

Figure S1 Flowchart of the inclusion process of the study.

Table S1 VIF for estimated muscle-related indices and age in age-adjusted models

Table S2 Numbers at risk, cumulative T2DM events, and censoring during follow-up

Table S3 Distribution of follow-up examination frequency

Appendix 2. Correlation analysis

Figure S2 Spearman's correlation of estimated muscle-related indices.

Table S4 Results of the correlation analysis of estimated muscle-related indices

Appendix 3. Kaplan-Meier survival analysis

Table S5 Tertile ranges of estimated muscle-related indices stratified by sex

Figure S3 Kaplan-Meier curves for cumulative incidence of T2DM by the tertile groups of estimated muscle-related indices. (A) ASM/height^2; (B) ASM/weight; (C) CCR; (D) pSMI.

Table S6 Number of participants at risk for Kaplan-Meier survival analysis at specified follow-up time points

Appendix 4. Cox regression analysis

Figure S4 Cox regression forest plot. **(A)** Continuous variables; **(B)** Categorical variable.

Table S7 FDR-adjusted P values for the primary associations

Table S8 Associations of estimated muscle-related indices with incident T2DM after additional adjustment for age and/or HbA1c

Appendix 5. Dose–response analysis

Table S9 RCS analyses of estimated muscle-related indices and incident T2DM

Appendix 6. Incremental discriminative value and internal validation

Table S10 Harrell's C-index of the baseline model and models with each estimated muscle-related index added

Table S11 Time-dependent AUCs of the prediction models at prespecified follow-up horizons

Table S12 Incremental discrimination of the estimated muscle-related indices assessed by

time-dependent ΔAUC

Table S13 Bootstrap optimism-corrected incremental discrimination of the estimated muscle-related indices

Figure S5 Bootstrap optimism-corrected time-dependent AUCs of the prediction models.

Figures S6–S8 Calibration plots of the prediction model (Model 3) at 3, 5, and 8 years.

Figures S9–S11 Calibration plots of the prediction model (Model 3 + age + HbA1c) at 3, 5, and 8 years.

Appendix 7. Subgroup analysis

Table S14 Sex interaction analyses between estimated muscle-related indices and incident T2DM

Table S15 Sex subgroup analysis of the association between estimated muscle-related indices and the risk of incident T2DM

Table S16 BMI interaction analyses between estimated muscle-related indices and incident T2DM

Table S17 BMI subgroup analysis of the association between estimated muscle-related indices and the risk of incident T2DM

Appendix 8. Sensitivity analysis

Table S18 Sensitivity analysis results of the association between estimated muscle-related indices and the risk of incident T2DM (excluding early incident T2DM cases)

Table S19 Sensitivity analysis using interval-censored survival models

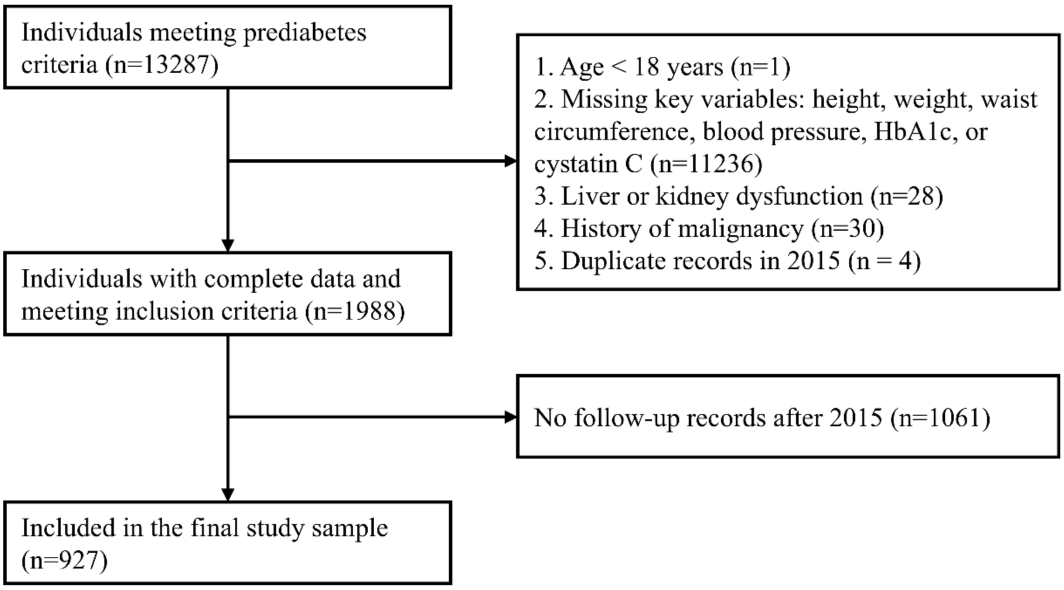

Figure S1 Flowchart of the inclusion process of the study.

Table S1 VIF for estimated muscle-related indices and age in age-adjusted models

Estimated muscle-related index	VIF of index	VIF of age
ASM/height^2	8.838	1.275
ASM/weight	8.773	1.275
CCR	1.215	1.275
pSMI	4.831	1.275

Abbreviation: ASM: appendicular skeletal muscle mass; CCR: Creatinine/Cystatin C Ratio; pSMI: predicted skeletal muscle mass index.

Table S2 Numbers at risk, cumulative T2DM events, and censoring during follow-up

Follow-up time	At risk	Cumulative T2DM events	Cumulative censored	Censoring rate
1 year	841	33	53	5.72%
3 years	552	114	261	28.16%
5 years	439	154	334	36.03%
8 years	280	189	458	49.41%
10 years	108	204	615	66.34%

Note: Participants at risk at each time point were those who had neither developed T2DM nor been censored before that time. Cumulative T2DM events and cumulative censored participants were calculated up to each prespecified time horizon. Because follow-up examinations were conducted at non-uniform intervals, these time points represent analytical horizons rather than scheduled examination dates.

Table S3 Distribution of Follow-up Examination Frequency

Follow-up examinations after baseline	*n*	%
1	312	33.66
2	177	19.09
3	92	9.92
4	84	9.06
⩾5	262	28.26

Note: The number of follow-up examinations refers to examinations conducted after the baseline examination during the available observation period.

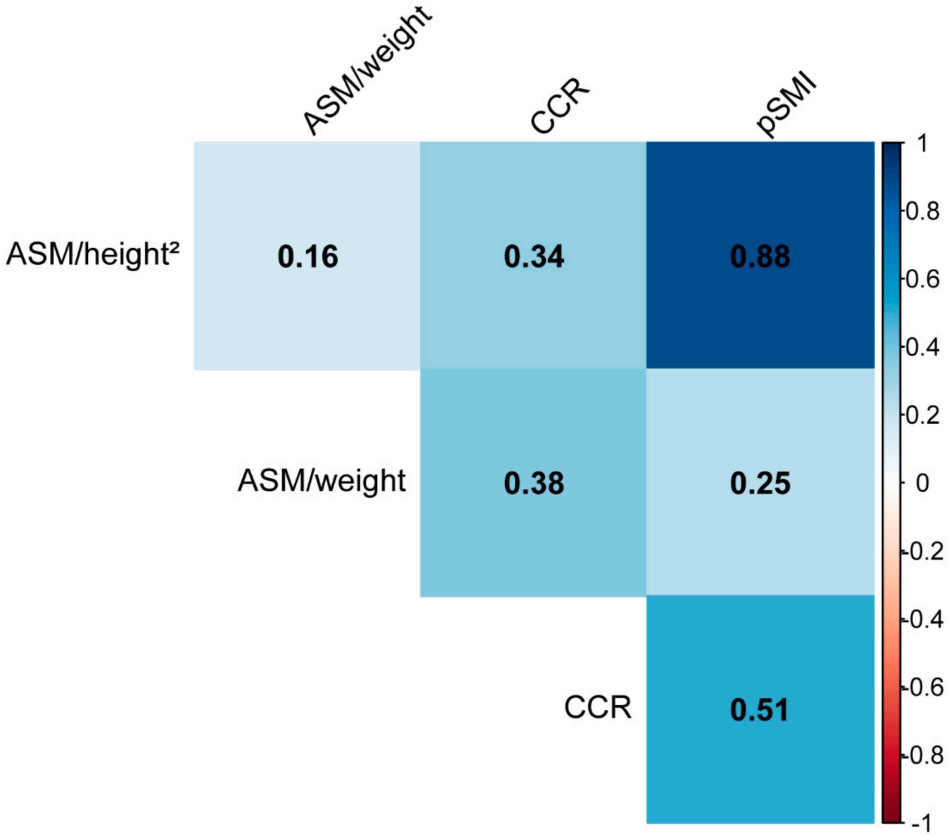

Figure S2 Spearman's correlation of estimated muscle-related indices.

Table S4 Results of the correlation analysis of estimated muscle-related indices

Index	ASM/height^2	ASM/weight	CCR	pSMI
**ASM/height^2**	1.000	0.161	0.337	0.883
**ASM/weight**		1.000	0.378	0.248
**CCR**			1.000	0.508
**pSMI**				1.000

Note: All correlations were significant with $p < 0.05$.

Table S5 Tertile ranges of estimated muscle-related indices stratified by sex

	ASM/height^2	ASM/weight	CCR	pSMI
**Male (*n* = 696)**				
Tertile 1 (*n* = 232)	6.59-7.92	0.28-0.32	0.49-0.78	6.19-7.89
Tertile 2 (*n* = 232)	7.92-8.35	0.32-0.33	0.78-0.89	7.89-8.41
Tertile 3 (*n* = 232)	8.35-10.47	0.33-0.40	0.89-2.68	8.41-11.35
**Female (*n* = 231)**				
Tertile 1 (*n* = 77)	4.70-5.81	0.23-0.26	0.36-0.64	5.35-6.28
Tertile 2 (*n* = 77)	5.81-6.35	0.26-0.27	0.64-0.74	6.28-6.68
Tertile 3 (*n* = 77)	6.35-8.75	0.27-0.31	0.74-1.30	6.68-8.77

Note: Tertile 1: Low group; Tertile 2: Medium group; Tertile 3: High group.

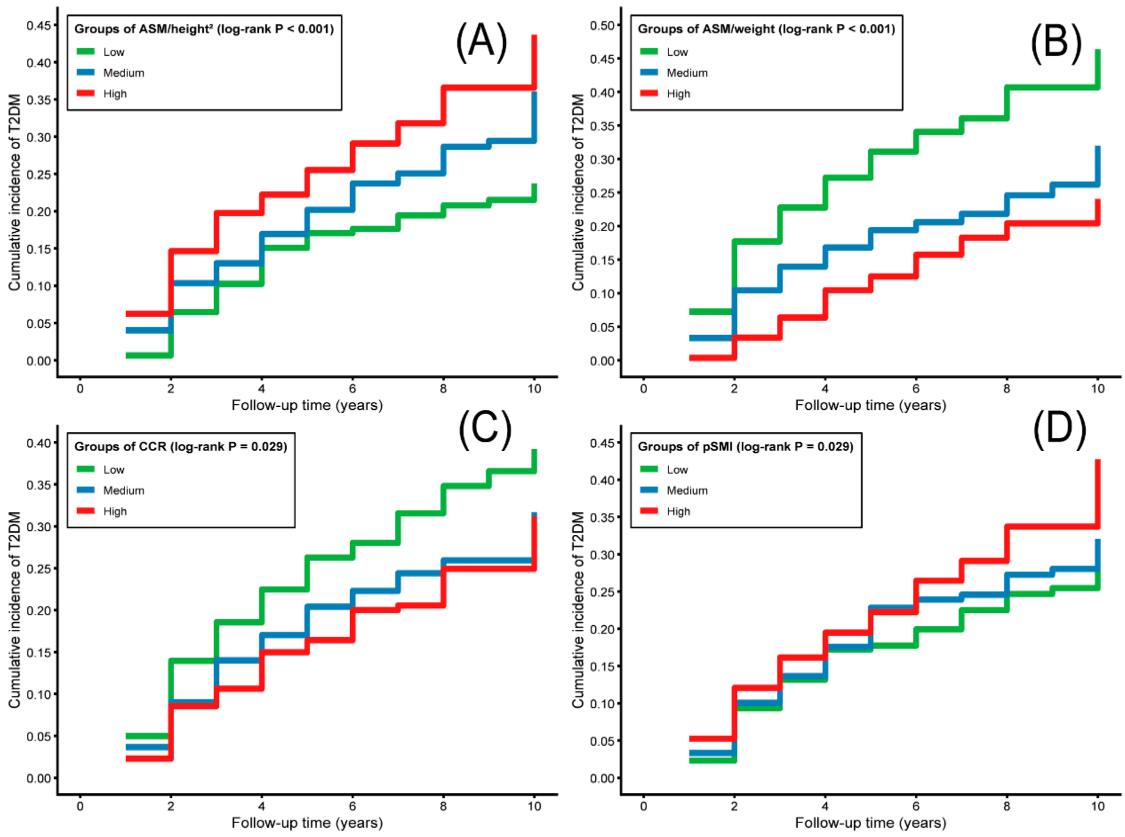

Figure S3 Kaplan-Meier curves for cumulative incidence of T2DM by the tertile groups of estimated muscle-related indices. (A) ASM/height^2; (B) ASM/weight; (C) CCR; (D) pSMI. **Note:** Participants were categorized into low, medium, and high groups based on gender-specific tertiles. The log-rank test was used to compare differences between groups. *P*-values are shown in each panel.

Table S6 Number of participants at risk for Kaplan-Meier survival analysis at specified follow-up time points

Index	Group	Time-0	Time-2	Time-4	Time-6	Time-8	Time-10
ASM/height^2	Low	309	233	179	144	112	48
ASM/height^2	Medium	309	212	156	118	92	33
ASM/height^2	High	309	193	146	108	76	27
ASM/weight	Low	309	190	134	102	75	25
ASM/weight	Medium	309	210	166	131	96	38
ASM/weight	High	309	238	181	137	109	45
CCR	Low	309	199	146	108	75	28
CCR	Medium	309	212	153	116	91	32
CCR	High	309	227	182	146	114	48
pSMI	Low	309	223	170	130	98	43
pSMI	Medium	309	212	160	125	99	36
pSMI	High	309	203	151	115	83	29

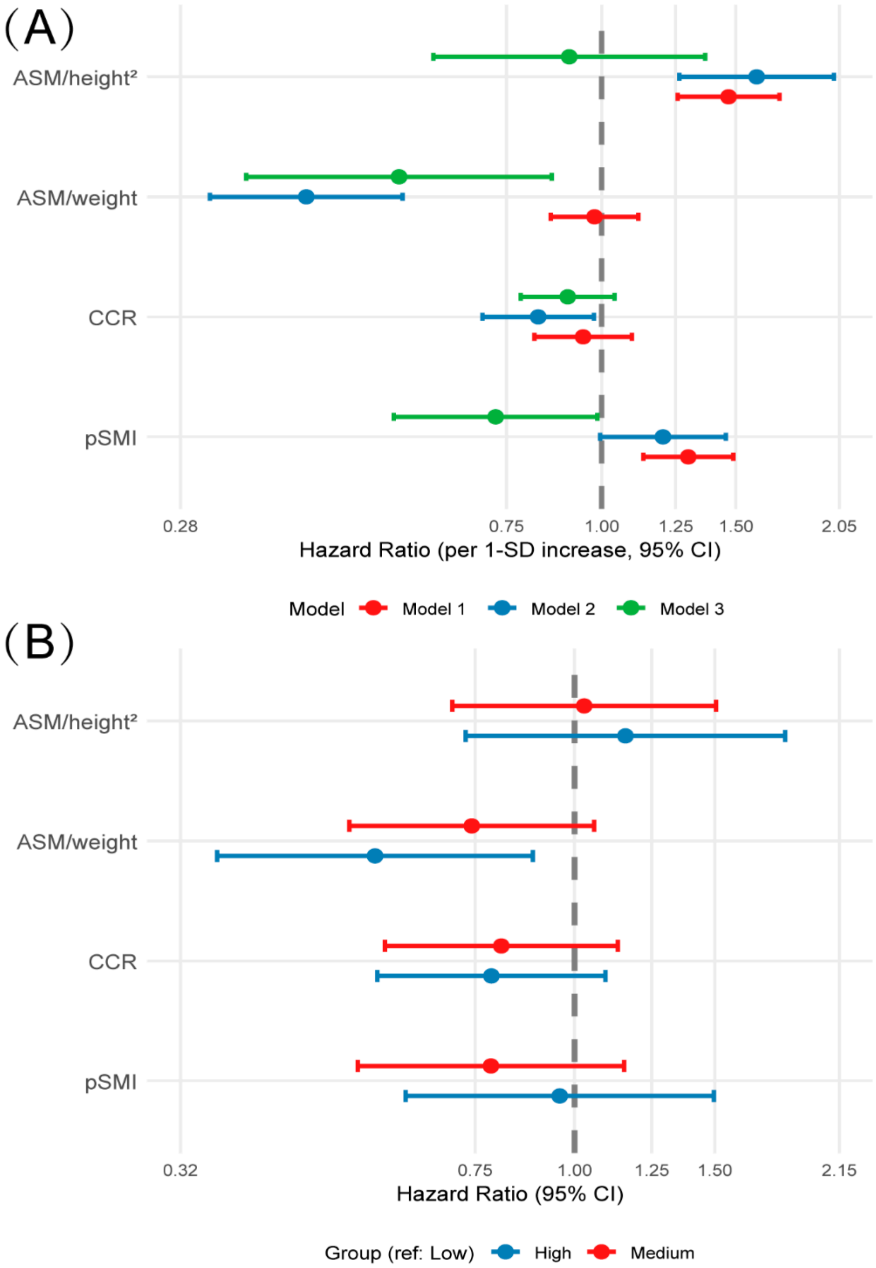

Figure S4 Cox regression forest plot. **(A)** Continuous variables. **(B)** Categorical variable. **Note:** The model was adjusted for sex, smoking, drinking, hypertension, WC, FBG, TGs, HDL-C, ALT, hs-CRP, and albumin.

Table S7 FDR-adjusted *P* values for the primary associations

**Index**	***p*-unadjusted**	***q*-FDR**
ASM/height^2	0.639	0.639
ASM/weight	0.009	0.036
CCR	0.155	0.207
pSMI	0.041	0.082

Note: *p* values were adjusted for multiple testing using the Benjamini–Hochberg false discovery rate procedure across the four primary associations evaluated in Model 3. An FDR-adjusted *q* value <0.05 was considered statistically significant.

Table S8 Associations of estimated muscle-related indices with incident T2DM after additional adjustment for age and/or HbA1c

Model	ASM/height^2		ASM/weight		CCR		pSMI	
	**HR (95% CI)**	***p***	**HR (95% CI)**	***p***	**HR (95% CI)**	***p***	**HR (95% CI)**	***p***
**Per 1-SD increase**								
Model 3 + Age	1.23 (0.79-1.94)	0.361	0.68 (0.42-1.11)	0.124	0.95 (0.82-1.09)	0.438	0.92 (0.65-1.30)	0.620
Model 3 + HbA1c	0.85 (0.55-1.31)	0.456	0.70 (0.43-1.14)	0.156	1.02 (0.87-1.19)	0.802	0.84 (0.62-1.14)	0.266
Model 3 + Age + HbA1c	0.99 (0.61-1.60)	0.963	0.79 (0.47-1.32)	0.366	1.04 (0.89-1.22)	0.610	0.94 (0.66-1.33)	0.724
**Tertile 3 vs Tertile 1**								
Model 3 + Age	1.35 (0.84-2.17)	0.214	0.69 (0.43-1.11)	0.124	0.88 (0.63-1.23)	0.448	1.23 (0.77-1.96)	0.395
Model 3 + HbA1c	1.25 (0.77-2.04)	0.370	0.72 (0.45-1.16)	0.179	0.92 (0.66-1.29)	0.638	1.20 (0.75-1.90)	0.445
Model 3 + Age + HbA1c	1.37 (0.83-2.26)	0.219	0.80 (0.49-1.32)	0.390	0.97 (0.69-1.35)	0.843	1.38 (0.85-2.23)	0.195

Table S9 RCS analyses of estimated muscle-related indices and incident T2DM

Index	Overall *p* value	Nonlinearity *p* value
**Model 3**		
ASM/height2	0.5133	0.2591
ASM/weight	0.0001	0.0215
CCR	0.0012	0.0023
pSMI	0.0452	0.6920
**Model 3 + Age**		
ASM/height2	0.5151	0.4828
ASM/weight	0.0471	0.0510
CCR	0.3966	0.2709
pSMI	0.8792	0.9171
**Model 3 + HbA1c**		
ASM/height2	0.7298	0.7883
ASM/weight	0.1098	0.1193
CCR	0.0897	0.0332
pSMI	0.4539	0.5699
**Model 3 + Age + HbA1c**		
ASM/height2	0.9405	0.7284
ASM/weight	0.2422	0.1537
CCR	0.0951	0.0441
pSMI	0.8246	0.6091

Note: Overall *p* values test the null hypothesis that all spline coefficients are simultaneously equal to zero, whereas nonlinearity *p* values test the null hypothesis that the association is linear. Restricted cubic spline models used three knots placed at the 10th, 50th, and 90th percentiles, with the median value as the reference.

Table S10 Harrell's C-index of the baseline model and models with each estimated muscle-related index added

Model	Harrell's C-index	ΔC-index
Base	0.7673	0
Base + ASM/height^2	0.7677	0.0004
Base + ASM/weight	0.7725	0.0052
Base + CCR	0.7684	0.0011
Base + pSMI	0.7719	0.0046

Table S11 Time-dependent AUCs of the prediction models at prespecified follow-up horizons

Model	Year-1	Year-3	Year-5	Year-8	Year-10
Base	0.779 (0.698-0.859)	0.792 (0.748-0.835)	0.797 (0.756-0.838)	0.791 (0.749-0.833)	0.768 (0.711-0.825)
Base + ASM/height^2	0.777 (0.696-0.857)	0.793 (0.749-0.836)	0.799 (0.758-0.840)	0.791 (0.748-0.833)	0.767 (0.710-0.824)
Base + ASM/weight	0.793 (0.715-0.870)	0.798 (0.755-0.842)	0.801 (0.760-0.841)	0.794 (0.751-0.836)	0.775 (0.718-0.831)
Base + CCR	0.781 (0.700-0.862)	0.793 (0.749-0.837)	0.800 (0.759-0.841)	0.793 (0.751-0.835)	0.772 (0.716-0.829)
Base + pSMI	0.781 (0.702-0.860)	0.802 (0.760-0.844)	0.805 (0.765-0.845)	0.795 (0.753-0.838)	0.770 (0.713-0.827)

Note: The C-index is expressed as a point estimate (95% CI). The variables adjusted for in the baseline model include: sex, smoking, drinking, hypertension, WC, FBG, TGs, HDL-C, ALT, hs-CRP, and albumin.

Table S12 Incremental discrimination of the estimated muscle-related indices assessed by time-dependent ΔAUC

Index	Year-1		Year-3		Year-5		Year-8		Year-10	
	**ΔAUC (95% CI)**	***p***	**ΔAUC (95% CI)**	***p***	**ΔAUC (95% CI)**	***p***	**ΔAUC (95% CI)**	***p***	**ΔAUC (95% CI)**	***p***
**Model 3**										
ASM/height^2	-0.0022 (-0.0056, 0.0013)	0.220	0.0008 (-0.0014, 0.003)	0.489	0.0018 (-0.0001, 0.0038)	0.068	-0.0002 (-0.0023, 0.0019)	0.838	-0.0015 (-0.0041, 0.001)	0.231
ASM/weight	0.0143 (-0.0001, 0.0286)	0.052	0.0066 (-0.0051, 0.0184)	0.268	0.0034 (-0.0072, 0.0141)	0.529	0.0026 (-0.0082, 0.0135)	0.634	0.0065 (-0.0081, 0.021)	0.385
CCR	0.0023 (-0.0043, 0.0089)	0.494	0.0015 (-0.0073, 0.0102)	0.744	0.0026 (-0.0042, 0.0094)	0.461	0.0022 (-0.0041, 0.0085)	0.495	0.0053 (-0.0039, 0.0125)	0.309
pSMI	0.0019 (-0.0073, 0.0112)	0.683	0.0103 (0.0007, 0.0199)	0.035	0.0081 (0.0003, 0.0160)	0.043	0.0042 (-0.0047, 0.0131)	0.352	0.0018 (-0.0111, 0.0147)	0.784
**Model 3 + Age + HbA1c**										
ASM/height^2	0.0000 (-0.0030, 0.0004)	0.846	0.0000 (-0.0003, 0.0003)	0.971	0.0001 (-0.0002, 0.0003)	0.474	0.0000 (-0.0003, 0.0004)	0.794	0.0001 (-0.0002, 0.0003)	0.570
ASM/weight	0.0038 (-0.0006, 0.0083)	0.088	0.0006 (-0.0020, 0.0033)	0.646	-0.0001 (-0.0028, 0.0025)	0.915	0.0004 (-0.0026, 0.0034)	0.778	0.0001 (-0.0041, 0.0042)	0.976
CCR	-0.0003 (-0.0022,	0.712	-0.0009 (-0.0023,	0.241	-0.0007 (-0.0021,	0.313	-0.0005 (-0.0021,	0.570	-0.0007 (-0.0028,	0.515

	0.0015)		0.0006)		0.0007)		0.0011)		0.0014)	
pSMI	-0.0002 (-0.0014, 0.0009)	0.712	0.0006 (-0.0004, 0.0016)	0.247	0.0005 (-0.0005, 0.0016)	0.327	0.0006 (-0.0007, 0.0019)	0.370	0.0014 (-0.0007, 0.0035)	0.178

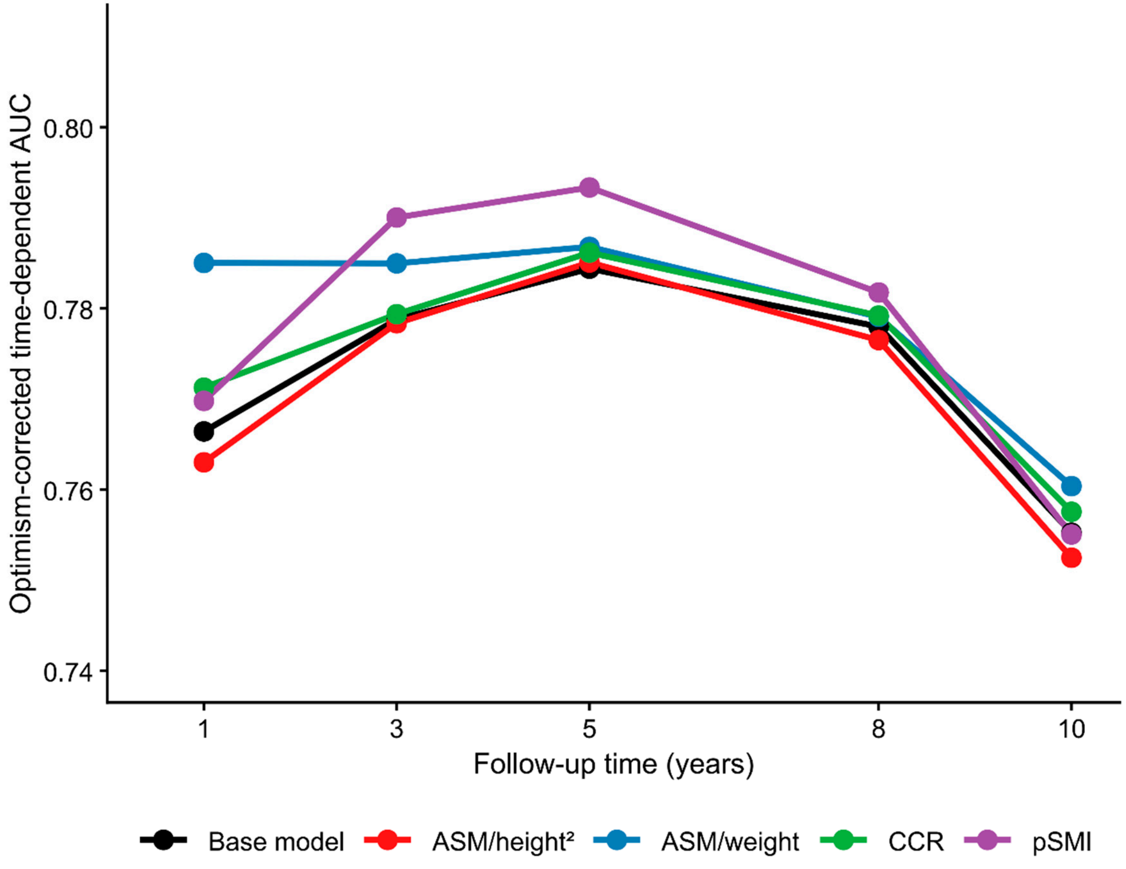

Figure S5 Bootstrap optimism-corrected time-dependent AUCs of the prediction models

Table S13 Bootstrap optimism-corrected incremental discrimination of the estimated muscle-related indices

Model	Time	Apparent-ΔAUC	Optimism-corrected ΔAUC (95% CI)	Bootstrap-*p*	Mean-optimism-ΔAUC
**Model 3**					
ASM/height^2	1	-0.0022	-0.003 (-0.013–0.010)	0.858	0.0012
	3	0.0008	-0.000 (-0.004–0.010)	0.722	0.0012
	5	0.0018	0.001 (-0.006–0.014)	0.72	0.0011
	8	-0.0002	-0.001 (-0.003–0.006)	0.778	0.0013
	10	-0.0015	-0.003 (-0.008–0.007)	0.96	0.0012
ASM/weight	1	0.0143	0.014 (-0.001–0.040)	0.068	0.0006
	3	0.0066	0.005 (-0.002–0.022)	0.144	0.0015
	5	0.0034	0.002 (-0.004–0.014)	0.396	0.0014
	8	0.0026	0.001 (-0.004–0.014)	0.48	0.0014
	10	0.0065	0.005 (-0.004–0.025)	0.296	0.0012
CCR	1	0.0023	0.002 (-0.005–0.016)	0.59	0.0004
	3	0.0015	-0.000 (-0.004–0.017)	0.54	0.0018
	5	0.0026	0.001 (-0.003–0.016)	0.402	0.0015
	8	0.0022	0.001 (-0.003–0.015)	0.404	0.0013
	10	0.0043	0.003 (-0.005–0.018)	0.514	0.0008
pSMI	1	0.0019	0.002 (-0.009–0.015)	0.77	0.0002
	3	0.0103	0.009 (0.0001–0.028)	0.048	0.0010
	5	0.0081	0.007 (-0.0002–0.022)	0.066	0.0008
	8	0.0042	0.003 (-0.002–0.017)	0.242	0.0008
	10	0.0018	0.001 (-0.006–0.022)	0.544	0.0011
**Model 3 + Age + HbA1c**					
ASM/height^2	1	0.000036	-0.001 (-0.006–0.006)	0.962	0.0006
	3	0.000005	-0.001 (-0.002–0.003)	0.942	0.0007
	5	0.00009	-0.001 (-0.004–0.005)	0.998	0.0008
	8	0.00004	-0.001 (-0.002–0.004)	0.914	0.0009

	10	0.00008	-0.001 (-0.005–0.007)	0.900	0.0009
ASM/weight	1	0.003846	0.004 (-0.005–0.014)	0.462	0.0003
	3	0.000626	-0.000 (-0.002–0.004)	0.880	0.0008
	5	-0.00014	-0.001 (-0.004–0.003)	0.746	0.0008
	8	0.00043	-0.000 (-0.002–0.005)	0.756	0.0008
	10	0.00006	-0.001 (-0.004–0.009)	0.770	0.0007
CCR	1	-0.00035	-0.001 (-0.005–0.004)	0.932	0.0003
	3	-0.00086	-0.002 (-0.004–0.004)	0.824	0.0008
	5	-0.00071	-0.002 (-0.004–0.004)	0.852	0.0009
	8	-0.00047	-0.001 (-0.004–0.004)	0.940	0.0008
	10	-0.00069	-0.002 (-0.006–0.007)	0.958	0.0008
pSMI	1	-0.00022	-0.000 (-0.004–0.003)	0.986	0.0003
	3	0.000583	-0.000 (-0.003–0.006)	0.820	0.0007
	5	0.000535	-0.000 (-0.002–0.004)	0.812	0.0007
	8	0.000611	-0.000 (-0.002–0.006)	0.768	0.0008
	10	0.001446	0.000 (-0.004–0.007)	0.808	0.0010
ASM/height^2	1	0.00004	-0.001 (-0.006–0.006)	0.962	0.0006
	3	0.000005	-0.001 (-0.002–0.003)	0.942	0.0007
	5	0.00009	-0.001 (-0.004–0.005)	0.998	0.0008
	8	0.00004	-0.001 (-0.002–0.004)	0.914	0.0009
	10	0.00008	-0.001 (-0.005–0.007)	0.900	0.0009

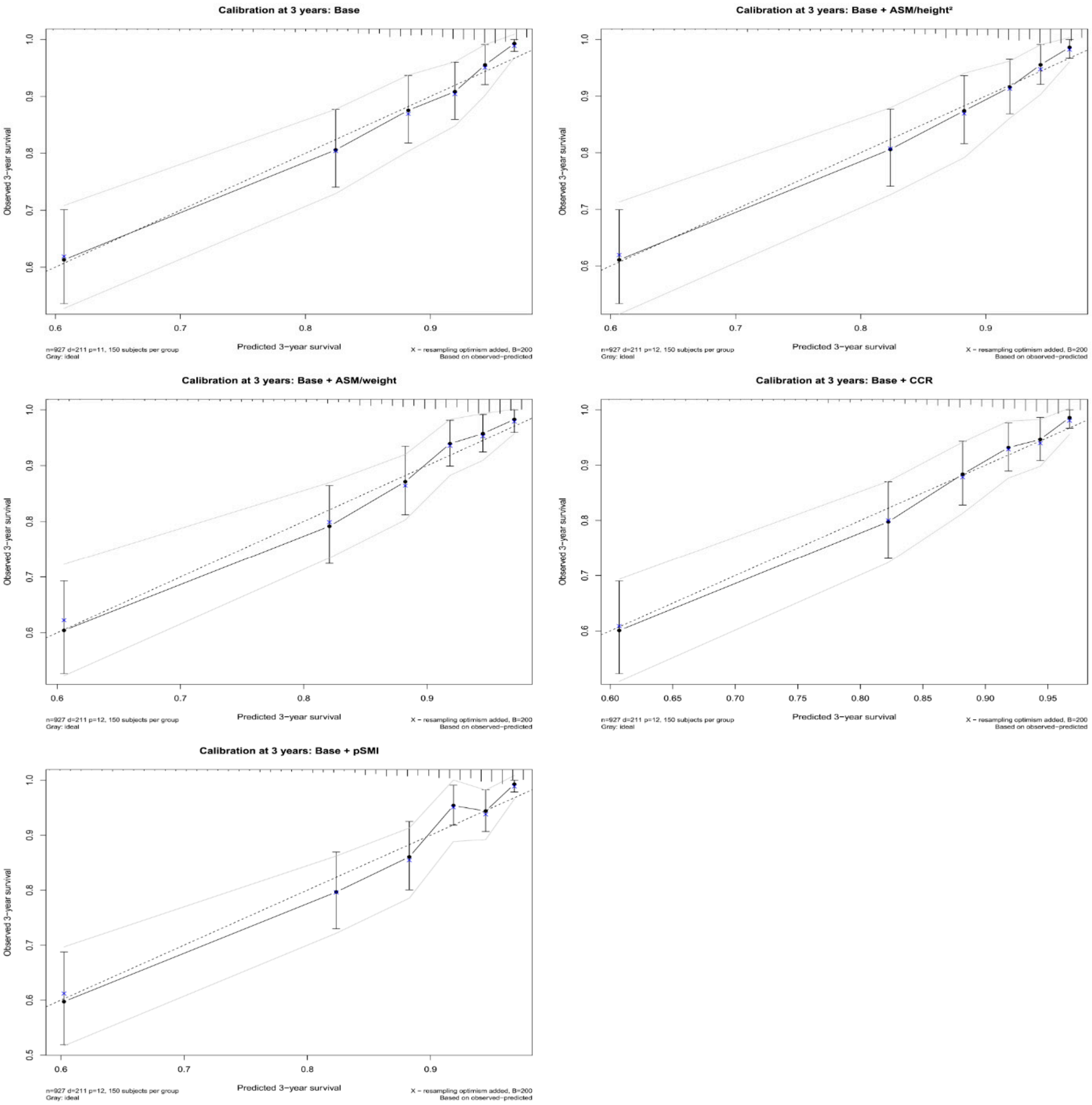

Figure S6 Calibration plots of the prediction model (Model 3) at 3 years

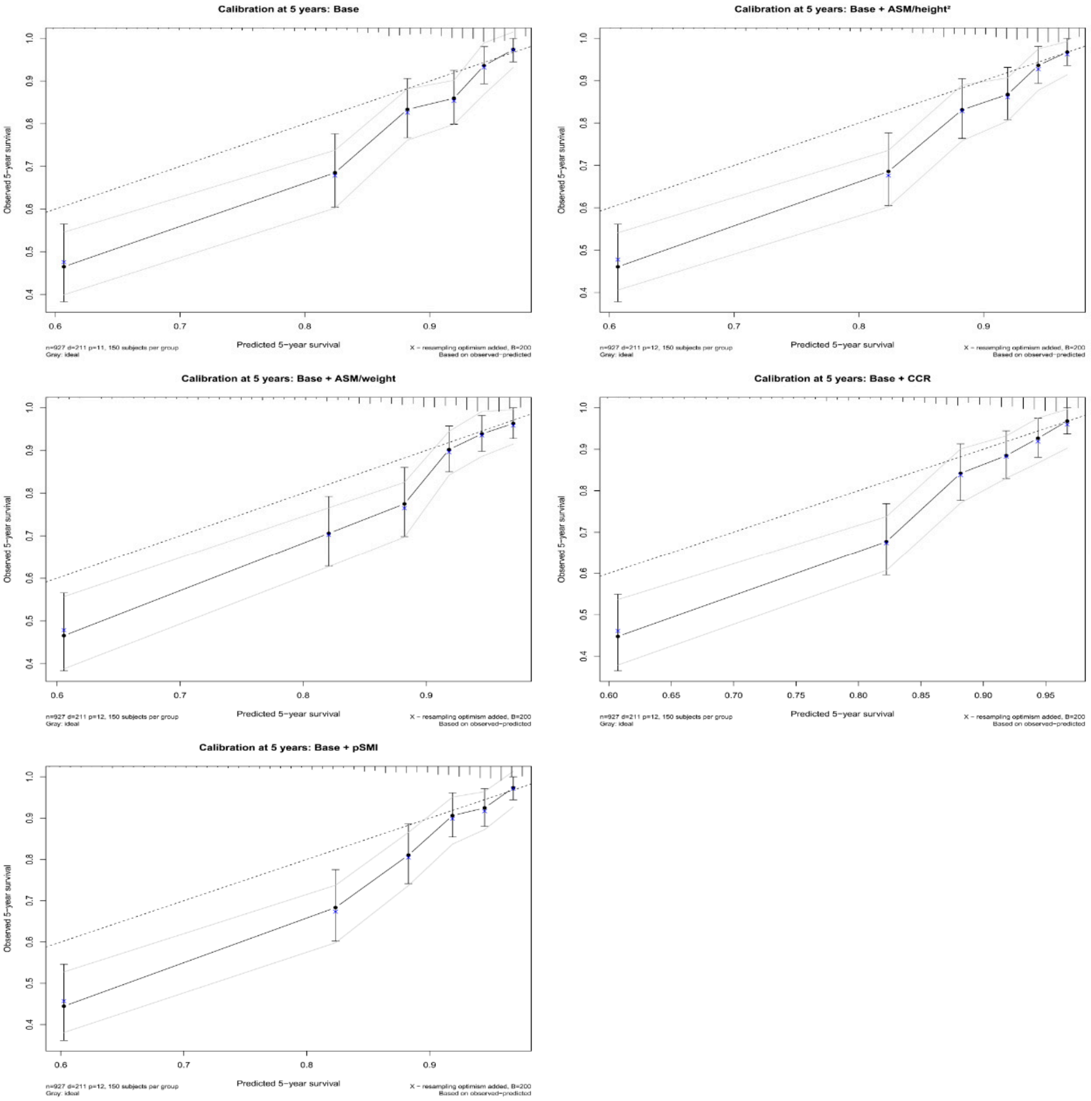

Figure S7 Calibration plots of the prediction model (Model 3) at 5 years

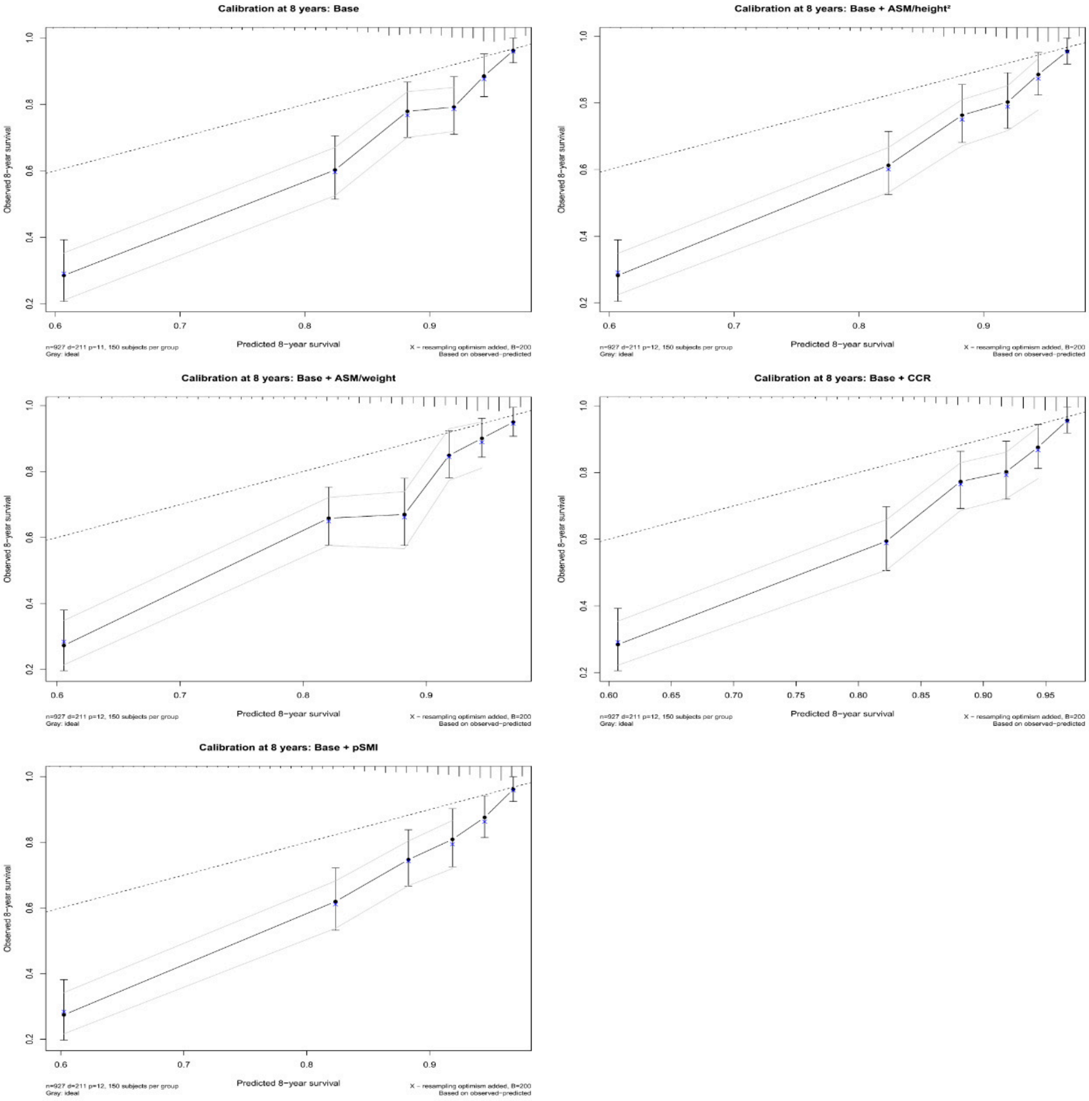

Figure S8 Calibration plots of the prediction model (Model 3) at 8 years

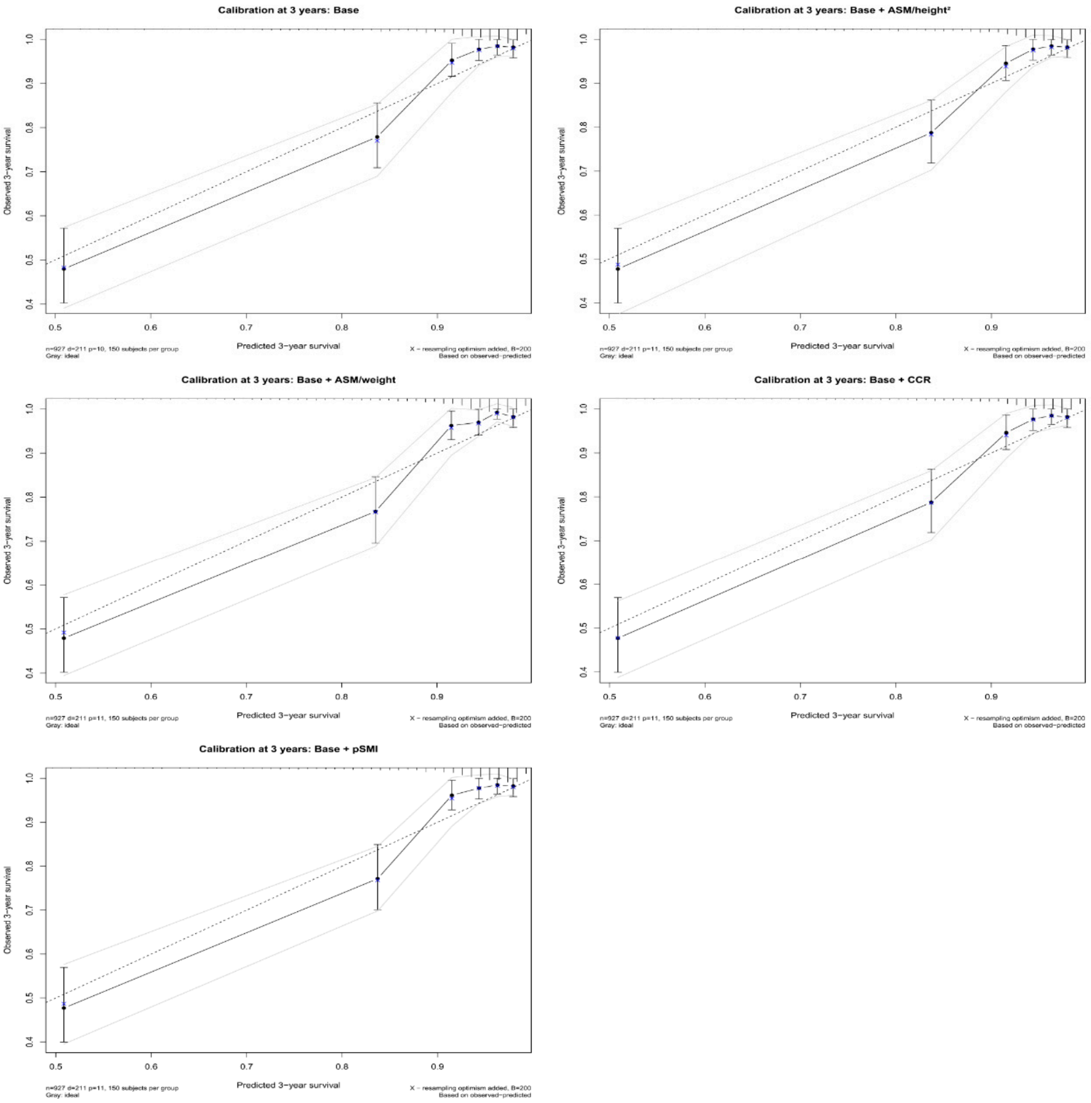

Figure S9 Calibration plots of the prediction model (Model 3 + Age + HbA1c) at 3 years

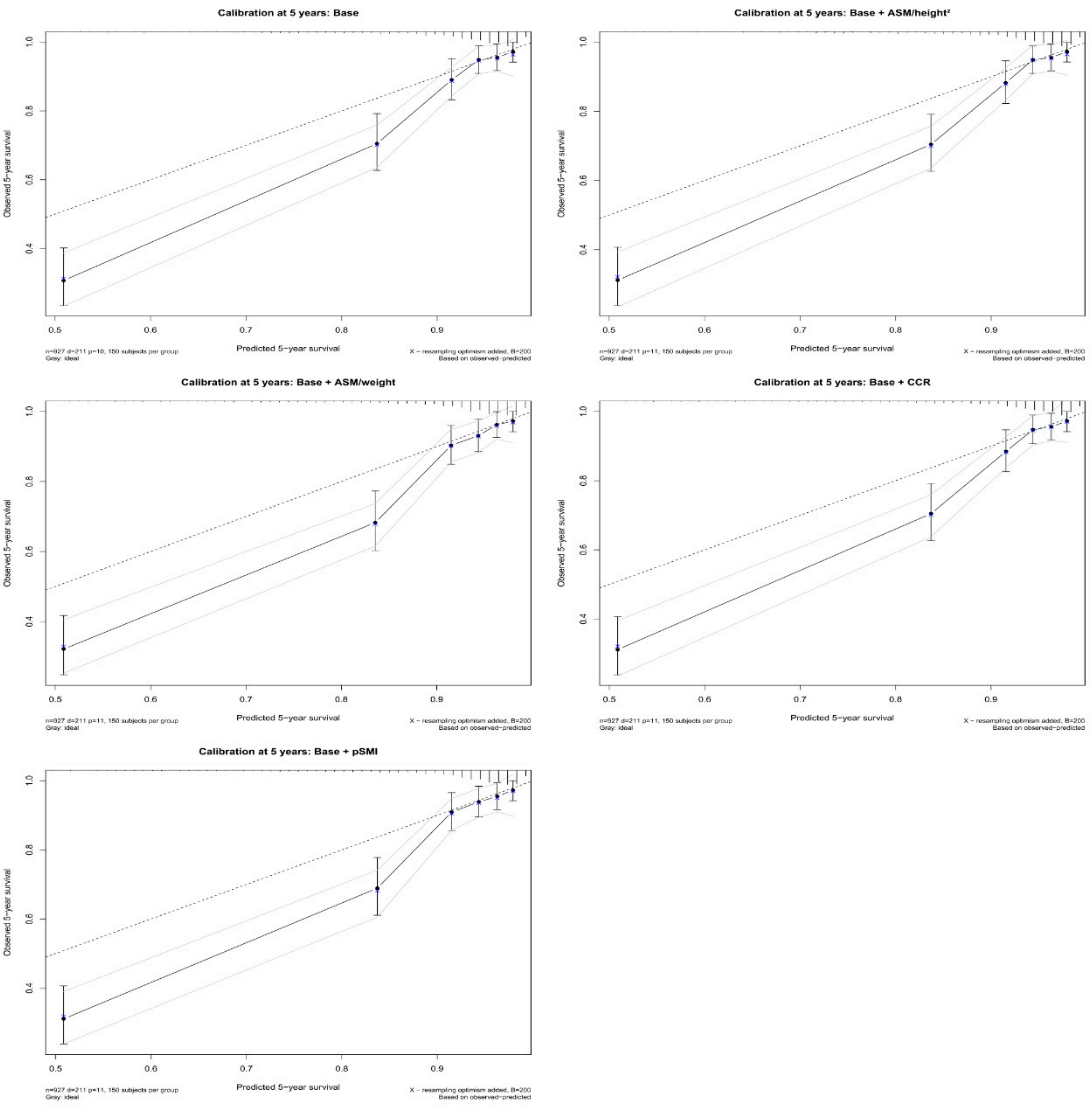

Figure S10 Calibration plots of the prediction model (Model 3 + Age + HbA1c) at 5 years

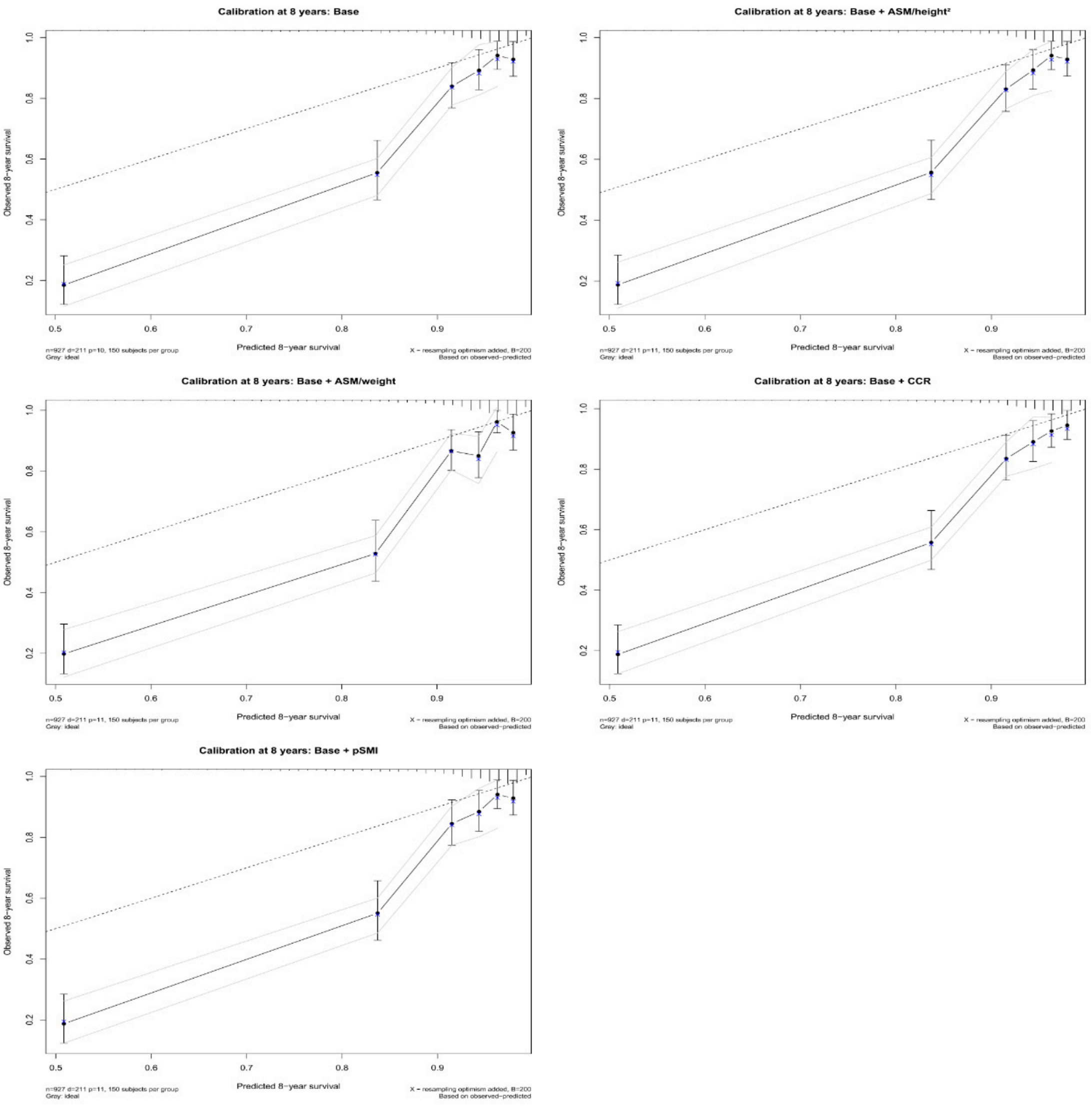

Figure S11 Calibration plots of the prediction model (Model 3 + Age + HbA1c) at 8 years

Table S14 Sex interaction analyses between estimated muscle-related indices and incident T2DM

Index	Interaction-HR (95% CI)	Interaction-*p*
**Model 3**		
ASM/height2	1.38 (0.72-2.65)	0.327
ASM/weight	0.19 (0.07-0.52)	0.001
CCR	0.35 (0.20-0.61)	< 0.001
pSMI	0.94 (0.49-1.78)	0.843
**Model 3+Age+HbA1c**		
ASM/height2	0.95 (0.52-1.73)	0.866
ASM/weight	0.32 (0.11-0.95)	0.041
CCR	0.33 (0.17-0.64)	0.001
pSMI	0.75 (0.42-1.33)	0.325

Table S15 Sex subgroup analysis of the association between estimated muscle-related indices and the risk of incident T2DM

Index	HR (95% CI)	*p*
**Male**		
ASM/height^2	1.02 (0.65-1.59)	0.942
ASM/weight	0.62 (0.38-1.02)	0.060
CCR	0.96 (0.84-1.10)	0.558
pSMI	0.84 (0.61-1.17)	0.308
**Female**		
ASM/height^2	0.47 (0.16-1.42)	0.181
ASM/weight	0.11 (0.03-0.38)	<0.001
CCR	0.31 (0.17-0.59)	<0.001
pSMI	0.28 (0.11-0.72)	0.009

Note: The effect size represents the corresponding HR (95% CI) for 1 SD increase in the muscle index. The model was adjusted for smoking, drinking, hypertension, WC, FBG, TGs, HDL-C, ALT, hs-CRP, and albumin.

Table S16 BMI interaction analyses between estimated muscle-related indices and incident T2DM

**Index**	**Comparison**	**Interaction-HR (95% CI)**	**Interaction-*p***
ASM/height2	Overweight	0.82 (0.52-1.29)	0.3878
	Obese	0.68 (0.31-1.50)	0.3397
ASM/weight	Overweight	0.78 (0.55-1.10)	0.1607
	Obese	0.67 (0.32-1.40)	0.2856
CCR	Overweight	0.97 (0.70-1.33)	0.8474
	Obese	1.10 (0.76-1.60)	0.6163
pSMI	Overweight	0.89 (0.59-1.33)	0.5644
	Obese	0.61 (0.33-1.12)	0.1134

Table S17 BMI subgroup analysis of the association between estimated muscle-related indices and the risk of incident T2DM

**Index**	**HR (95% CI)**	***p***
**Normal weight (n = 384)**		
ASM/height^2	0.74 (0.28-1.92)	0.534
ASM/weight	0.22 (0.09-0.56)	0.002
CCR	0.79 (0.61-1.03)	0.084
pSMI	0.71 (0.41-1.22)	0.211
**Overweight (n = 434)**		
ASM/height^2	1.04 (0.44-2.43)	0.931
ASM/weight	0.41 (0.17-0.96)	0.041
CCR	0.90 (0.71-1.14)	0.389
pSMI	0.98 (0.66-1.46)	0.932
**Obese (n = 109)**		
ASM/height^2	0.90 (0.21-3.83)	0.889
ASM/weight	0.99 (0.16-6.04)	0.995
CCR	0.79 (0.51-1.23)	0.298
pSMI	0.48 (0.24-0.96)	0.039

Note: The underweight group (n = 17) was merged into the normal-weight group because no T2DM events occurred, resulting in a final normal-weight group of 384 participants. The effect size represents the corresponding HR (95% CI) for 1 SD increase in the muscle index. The model was adjusted for sex, smoking, drinking, hypertension, FBG, TGs, HDL-C, ALT, hs-CRP, and albumin.

Table S18 Sensitivity analysis results of the association between estimated muscle-related indices and the risk of incident T2DM

	**ASM/height^2**		**ASM/weight**		**CCR**		**pSMI**	
	**HR (95% CI)**	***p***	**HR (95% CI)**	***p***	**HR (95% CI)**	***p***	**HR (95% CI)**	***p***
Per 1-SD increase	0.79 (0.50-1.23)	0.295	0.61 (0.37-1.00)	0.051	0.93 (0.81-1.07)	0.371	0.71 (0.51-0.99)	0.049
Tertile 1 (reference)	1	-	1	-	1	-	1	-
Tertile 2	0.90 (0.60-1.34)	0.595	0.80 (0.54-1.18)	0.252	0.81 (0.55-1.17)	0.255	0.77 (0.51-1.16)	0.209
Tertile 3	0.99 (0.60-1.63)	0.966	0.67 (0.41-1.08)	0.101	0.82 (0.57-1.17)	0.267	0.92 (0.57-1.49)	0.736
P value for trend		0.961		0.099		0.266		0.804

Note: Thirty-three participants who developed T2DM within the first year of follow-up were excluded, leaving 894 participants and 178 T2DM events for the sensitivity analysis. The model was adjusted for sex, smoking, drinking, hypertension, WC, FBG, TGs, HDL-C, ALT, hs-CRP, and albumin.

Table S19 Sensitivity analysis using interval-censored survival models

Index	Cox HR (95% CI)	*p*	Log-logistic HR (95% CI)	*p*
**Model 3**				
ASM/height^2	0.92 (0.61-1.38)	0.679	0.92 (0.61-1.38)	0.677
ASM/weight	0.54 (0.34-0.86)	0.010	0.54 (0.34-0.86)	0.009
CCR	0.91 (0.79-1.05)	0.192	0.91 (0.79-1.05)	0.193
pSMI	0.75 (0.55-1.03)	0.072	0.75 (0.55-1.03)	0.072
**Model 3+Age+HbA1c**				
ASM/height^2	0.91 (0.55-1.51)	0.719	0.82 (0.48-1.38)	0.450
ASM/weight	0.82 (0.48-1.39)	0.456	0.91 (0.55-1.51)	0.722
CCR	1.05 (0.90-1.22)	0.517	1.05 (0.90-1.22)	0.514
pSMI	0.98 (0.68-1.40)	0.892	0.98 (0.68-1.40)	0.897

Note: For participants who developed T2DM, the event was considered to occur within the interval between the last examination without T2DM and the first examination meeting the diagnostic criteria. Model 3 was adjusted for smoking, drinking, hypertension, WC, FBG, TGs, HDL-C, ALT, hs-CRP, and albumin. The sensitivity model additionally adjusted for age and HbA1c.
